# Critical Role of Regrowth Conditions in Post-Cryopreservation of In Vitro Plant Germplasm

**DOI:** 10.3390/biology12040542

**Published:** 2023-04-02

**Authors:** Elena Popova, Irina Kulichenko, Haeng-Hoon Kim

**Affiliations:** 1 K.A. Timiryazev Institute of Plant Physiology of Russian Academy of Sciences, Botanicheskaya 35, Moscow 127276, Russia; 2 Department of Agricultural Life Science, Sunchon National University, Suncheon 57922, Republic of Korea

**Keywords:** antioxidant, ammonium, cryopreservation, embryogenic cultures, medium composition, oxidative stress, plant cell culture, plant growth regulators, regeneration, root culture, shoot tips

## Abstract

**Simple Summary:**

Optimization of regrowth conditions is critical for the successful cryopreservation of in vitro plant germplasm. This review discusses the five major strategies available at the regrowth stage for improving plant material performance after cryopreservation. These strategies involve physical factors (modification of osmotic environment, light/dark conditions, and light quality) and chemical factors (recovery medium composition, application of exogenous additives, and the influence of plant growth regulators depending on the type of cryopreserved materials). This summary is meant to serve as a guideline for choosing the most suitable regrowth conditions for plant germplasm to be cryopreserved. We also propose the combinations of factors that may benefit the recovery of cryopreservation-sensitive species and types of materials.

**Abstract:**

Cryopreservation is an effective option for the long-term conservation of plant genetic resources, including vegetatively propagated crops and ornamental plants, elite tree genotypes, threatened plant species with non-orthodox seeds or limited seed availability, as well as cell and root cultures useful for biotechnology. With increasing success, an arsenal of cryopreservation methods has been developed and applied to many species and material types. However, severe damage to plant material accumulating during the multi-step cryopreservation procedure often causes reduced survival and low regrowth, even when the optimized protocol is applied. The conditions at the recovery stage play a vital role in supporting material regrowth after cryopreservation and, when optimized, may shift the life-and-death balance toward a positive outcome. In this contribution, we provide an overview of the five main strategies available at the recovery stage to improve post-cryopreservation survival of in vitro plant materials and their further proliferation and development. In particular, we discuss the modification of the recovery medium composition (iron- and ammonium-free), exogenous additives to cope with oxidative stress and absorb toxic chemicals, and the modulation of medium osmotic potential. Special attention is paid to plant growth regulators used at various steps of the recovery process to induce the desired morphological response in cryopreserved tissues. Given studies on electron transport and energy provision in rewarmed materials, we discuss the effects of light-and-dark conditions and light quality. We hope that this summary provides a helpful guideline and a set of references for choosing the recovery conditions for plant species that have not been cryopreserved. We also propose that step-wise recovery may be most effective for materials sensitive to cryopreservation-induced osmotic and chemical stresses.

## 1. Introduction

Cryopreservation, i.e., the storage of viable material in liquid nitrogen (LN, −195.8 °C) or its vapor phase (−150~−195 °C), combined with in vitro technologies, offers a valuable option for the long-term conservation of plant species that are vegetatively propagated, with non-orthodox or limitedly available seeds [1,2,3,4]. In response to the urgent need for secure long-term conservation, collections of cryopreserved plant genetic resources have been established worldwide with capacities ranging from dozens to thousands of samples [5,6,7,8,9,10], and an increasing number of genebanks, botanic gardens, and scientific institutes have created cryobanks for their research and practical needs [11].

Cryopreservation involves freezing and storing plant materials at cryogenic temperatures, their rewarming, and revitalization, followed by the regenerating of plants or tissues of the preserved genotypes. Cryopreservation has been applied to diverse plant materials: seeds, pollen, dormant buds, shoot tips or axillary buds of in vitro plants, hairy or adventitious roots, embryonic axes, embryogenic and non-embryogenic cell cultures, protoplasts, microtubers, rhizome buds, etc. [1,12,13,14,15,16,17,18]. Establishing an in vitro culture system is often inevitable for the regeneration of cryopreserved propagules and the multiplication of plant material for study, conservation, and restoration purposes [14,19]. However, there are examples of successful cryopreservation studies where the use of in vitro culture was minimized or bypassed, e.g., cryopreserved seeds could be germinated ex vitro in an appropriate substrate [20,21], dormant buds of fruit trees were recovered via micrografting [17,22], pollen after cryopreservation could be used for pollination and fruit production [23]. However, in vitro-derived materials are most useful for the cryopreservation of vegetatively propagated staple crops, ornamental varieties, or elite tree genotypes [13,14,24]. Cryopreservation of in vitro propagules may also help conserve threatened species without the available seeds for banking or with recalcitrant seeds that cannot be preserved as embryos [2,3]. Moreover, cryopreservation is the only reliable option for the long-term conservation of biotechnological collections of isolated cell and root cultures that exist only in vitro [15,16,25,26].

**Figure 1:**
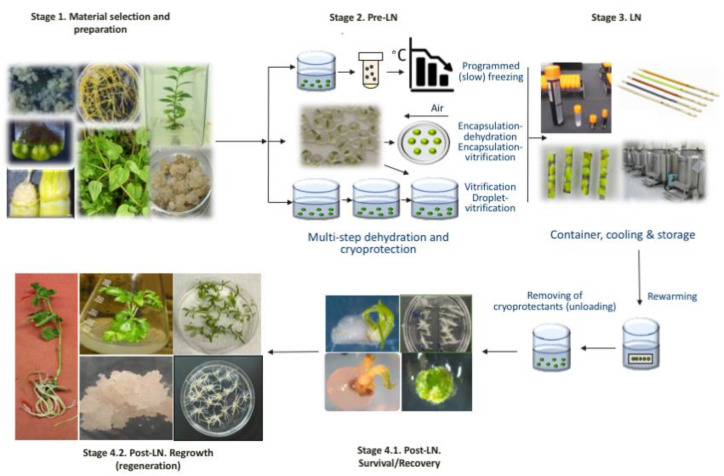
Schematic illustration of the cryopreservation process for in vitro plant germplasm.

Since its early days, plant cryopreservation was meant for a practical application in the long-term conservation of plant germplasm [27,28]. Still, its development is closely linked with advances in the fundamental knowledge of biophysics (ice crystallization, cryoprotection, low-temperature phase transition in complex solutes), the nature of freezing injuries in living tissues, and the mechanisms underlying plant freeze- and dehydration-tolerance [11,29]. With the formulation of major principles in plant cryopreservation and successful tests of a growing number of species, it became evident that there is no uniform method applicable through a wide range of plant taxa for ensuring the recovery of different types of materials. Although disappointing, this has resulted in an arsenal of different methods and cryopreservation protocols that can be further tailored to specific materials or individual genotypes with relatively few modifications (Figure 1) [10,19,30,31,32].

Preparation of donor (stock) plant material (Stage 1 in Figure 1) is vital to the success of cryopreservation. Multiple studies have investigated the effects of cold-hardening, donor plant age, and the selection of appropriate explants on the recovery after cryopreservation, as previously reviewed [11,13,18,19,24].

The diversity of cryopreservation methods (Stage 2. Pre-LN in Figure 1), their pros and cons, and their applicability to different plant materials have been reviewed in detail [1,11,19]. All cryopreservation methods are based on the sufficient dehydration of plant material at the pre-LN steps to avoid lethal intracellular ice crystallization. Most methods involve preliminary treatment with step-wise increasing concentrations of cryoprotectants (CPA) as a necessary step before LN exposure. The methods are differentiated by how the material is dehydrated, the type and concentration of CPA solutions employed, and the duration and temperature of CPA treatment [11,33].

The rate of cooling and rewarming provided by different carriers (cryoplates, aluminum foil strips, cryo-tubes, straws) is another important factor to consider (Stage 3. LN in Figure 1). Aluminum foils and cryoplates are primarily used in droplet-vitrification and cryoplate methods, respectively, due to fast heat transfer between the specimens and cooling or rewarming environment [14,19]. Cryo-tubes are employed in programmed freezing and vitrification methods when the samples are cryopreserved in a CPA mixture, and to cryopreserved explants embedded in alginate beads (encapsulation methods) [18,24,30].

Following the storage in cryotanks, materials are rapidly rewarmed to avoid dangerous ice recrystallization. Upon rewarming, the process is repeated in reverse: the cryoprotectants are washed-off, and explants are allowed to regenerate under controlled conditions (Stages 4.1 and 4.2. Post-LN in Figure 1).

Therefore, all modern cryopreservation methods are multi-stage procedures. The successful cryopreservation includes (1) adequately selected and prepared plant material, (2) optimized cryopreservation protocol (preculture, osmoprotection, cryoprotection with vitrification solution or encapsulation and air dehydration), (3) appropriate container, safe LN storage, rewarming and removing of CPA, and 4) recovering under the most favorable conditions and post-storage evaluation (Figure 1). The impact of each protocol stage on the success of cryopreservation has been previously reviewed [10,14,19,30,33]. All steps involve careful and precise manipulation of fragile and tiny explants and require the high operational skill of the personnel [7,14].

The majority of studies in plant cryopreservation are traditionally focused on the optimization of stage 2 (preculture conditions, duration and temperature of CPA treatment, and the composition of the CPA mixture) and stage 3 (containers, the concentration of unloading solution). This focus is logical as if the samples are lethally injured during the cryopreservation *per se*, they will not be able to recover, irrespective of how good the recovery conditions are. However, the situation is slowly changing. The progress in the fundamental understanding of cryopreservation-associated injuries led to more adaptive protocols [34]. Combined with a multifactorial experiment design or a systematic approach, it allowed a more significant variety of species to survive the extreme conditions of cryogenic storage [1,2,24,32,34]. Optimizing the recovery stage 4 (post-LN) is becoming increasingly important, with more species surviving after LN exposure.

Plant tissues always experience severe stress during cryopreservation and, after rewarming, require special care and a “nourishing” environment to recover and eventually produce new tissues. Regrettably, the importance of the post-LN stage is often overlooked, and the recovery conditions simply mimic those used at the propagation or pre-LN steps. However, some materials require special culture conditions (altered medium composition, specific growth regulators) and changes to the surrounding physical environment (darkness, gradual change in medium osmotic potential, etc.) to accomplish the recovery process [18,24]. The recovery stage becomes critical when materials are cryopreserved through non-optimized protocols and therefore are weakened from multiple injuries. A proper recovery medium is also essential when the in vitro conditions are not fully optimized (e.g., for the endangered species), or for recalcitrant genotypes that do not respond well to standard procedures developed for the same or relative taxa.

Below, we give an overview of the conditions that have proven crucial at the recovery stage and their impact on the regrowth of different types of in vitro plant material after cryopreservation.

## 2. Main Injuries in Plant Tissues during Cryopreservation and Five Strategies to Overcome Them at the Recovery Stage

### 2.1. Main Injuries in Plant Tissues during Cryopreseervation

Cryopreservation is considered successful when it ends with the growth and propagation of the cryopreserved materials. For example, after cryopreservation, meristematic tissues (buds, shoot tips) should develop plants with stem elongation, leaf formation, rooting, and a functional apex without signs of hyperhydration or abnormal morphology [11,14,24]. Fully recovered cell cultures should be capable of embryogenesis or retention of their growth and biosynthetic characteristics [15,25,26,35]. The issues related to the genetic and metabolic stability and field performance of plant materials after cryopreservation have been comprehensively reviewed [36].

The frequent use of the terms “survival”, “regrowth”, and “regeneration” to measure the success of cryopreservation can be misleading and require definition. “Survival” is now generally used to indicate initial changes in plant propagules after cryopreservation observed as the appearance or sustaining of green color, size increase, development of unorganized tissues, or spontaneous production of leaves or roots. These changes do not always lead to the subsequent development and formation of healthy plants or embryogenic tissues, or the re-initiation of cell suspension (depending on the desired outcome). “Regeneration”, in the context of cryopreservation, indicates the development of normal plantlets with stem, leaves, and roots. “Regrowth” may have the same meaning as “regeneration” but with a broader sense and may be used, depending on the nature of the cryopreserved material, to reflect the proliferation of embryogenic or undifferentiated cell culture, production of new protocorm-like bodies or adventitious rhizomes, etc. Simply put, “regrowth” indicates that plant material recovered after cryopreservation has fully retained its specific traits and is capable to growth and propagation.

Due to the specifics of cryopreservation, regrowth and regeneration are often lower than survival [33], which may negatively impact the costs and benefits of cryobanking programs, particularly on a large-scale [11,28]. Most of the world’s genebanks use the 40% plant regeneration threshold for cryopreservation to be considered adequate [5,7,9,28]. Probability tools were developed linking survival/regrowth percentage and the number of samples stored in the cryotank [37,38].

Even well-optimized protocols rarely permit 100% regrowth of in vitro plant germplasm after freezing and rewarming due to a wide range of cell injuries occurring during the process [33]. These injuries include potential mechanical damage by extracellular ice (in the programmed/slow freezing method), osmotic stress due to severe dehydration and application of highly concentrated CPA mixtures, increasing cytoplasm viscosity, and accumulating toxic solutes. These factors, acting collectively with cytotoxicity of high doses of CPA, result in extensive damage to cryopreserved tissues that have been reviewed in detail [33,39,40]. The ultrastructural studies of plant material at various stages of cryopreservation revealed significant changes in the intracellular integrity or organization of cells, damage to membrane structures, including plasmalemma, cell lysis, fragmentation of intracellular compartments, etc. [40,41,42]. Extensive stress leads to the boost of the reactive oxygen species (ROS) and the rapid development of oxidative stress (discussed below). Several studies revealed significant changes in plant tissue transcriptomic and proteomic profiles associated with various cryopreservation steps, including recovery [43,44,45,46]. In *Arabidopsis thaliana* shoot tips, the authors identified 180 transcripts that changed expression in response to cryoprotection and LN treatments, of which 67 were related to stress, defense, wounding, lipid, carbohydrate, abscisic acid, oxidation, temperature (cold/heat), or osmoregulation [45]. Carbon and energy metabolism-related proteins were the most substantially altered after cryopreservation of the protocorm-like bodies of *Dendrobium nobile* orchid [47]. Whelehan et al. [40] concluded that cryopreservation-induced cell damages significantly negatively impact energy metabolism, particularly reducing mitochondria functionality. The adenosine triphosphate (ATP) concentration in potato shoot tips changed on average from 28.9 nmol g^−1^ fresh weight (FW) to 5.6 nmol g^−1^ FW as a result of the cryopreservation procedure [48].

Nearly all cryopreservation methods induce DNA methylation in plant tissues [36]. Moreover, when meristematic propagules are cryopreserved through a non-optimized protocol, large zones of the apical dome may be destroyed, and plant regeneration is mediated by callus formation [35]. This indirect (callus-mediated) regeneration imposes the risk to genetic integrity and, eventually, may lead to the loss of essential cultivar traits [14,19,36].

### 2.2. Five Strategies at Recovery Stage to Overcome Cryopreservation-Induced Injuries

The expansive nature of cryopreservation-induced injuries suggest that, at the recovery stage, cells should be provided with a supportive environment to rapidly repair the reversible damages to cellular components, restore normal cell functioning, and regain the capability to proliferate and produce new tissues. This process is energy intensive [40] and is often “the race against time”, given the rapidly accumulating ROS and toxic chemicals in the injured specimens. Therefore, the conditions at the recovery stage should serve the following purposes:

- Provide optimum conditions for recovery without posing extra stress to already damaged plant materials;
- Remove toxic chemicals (residues of CPA, toxic substances released from the damaged cells, phenolic compounds, etc.);
- Provide easy access to nutrients since the connection between cell clusters in the rewarmed samples may be broken or temporarily disrupted;
- Support/activate repair mechanism to cope with damage to cellular structures, particularly membranes, lipid peroxidation, ROS accumulation, etc.;
- Support energy function through appropriate carbohydrate sources;
- Support the desired morphogenic or embryogenic response, e.g., direct regrowth from meristem-containing propagules, embryo development, new root formation, etc.

To better understand the specific requirements at the recovery step and rationalize, whenever possible, the effects of individual factors on the recovery of in vitro propagules, we analyzed over 180 papers on the cryopreservation of different plant materials published between the 1980s and 2022, as well as recent reviews on various aspects of plant cryopreservation. Maximum attention has been paid to papers describing original cryopreservation protocols and studies that mentioned modifications of the recovery stage to improve regrowth. We relied on our experience in plant cryobiology and the knowledge of the plant cryopreservation landscape to select the most representative papers for each factor investigated. Based on the digested information, we outlined five main strategies that can be applied, individually or in combination, throughout the recovery process for better results:

**Strategy 1. Modification of the osmotic environment.** This includes the step-wise transfer of rewarmed and washed from CPA materials to medium with a gradually decreasing sucrose concentration. The main goal is to avoid osmotic shock in plant tissues that are brought from highly concentrated CPA solutions to standard medium (3–5% sucrose). Another important factor is a physical state of the recovery medium.

**Strategy 2. Modification of medium composition.** Some nutrient medium components commonly used during plant propagation and maintenance in vitro may become toxic for cryopreserved materials and should be excluded at the recovery stage.

**Strategy 3. Exogenous additives.** These compounds are not the standard components of the culture medium but may be added specifically after cryopreservation to help cells cope with the extensive stress and recover. The compound groups often include enzymatic and non-enzymatic antioxidants, adsorbents that remove toxic chemicals released from dead tissues, and substances with proliferation-inducing activity.

**Strategy 4. Modification of plant growth regulators (PGRs).** The effect of PGRs during the recovery process is not yet well understood. Some PGRs are preferable after cryopreservation and some should be avoided. The composition of PGRs in recovery medium depends on the type of plant material and may be altered in the course of regrowth process.

**Strategy 5. Light/dark conditions and light quality.** Light is involved in plant energy provision and triggers specific signaling cascades. The optimum dark/light combination at various stages of recovery is vital for the normal development of cryopreserved tissues.

These five strategies are discussed below in detail.

## 3. Strategy 1. Osmotic Environment

### 3.1. Step-Wise Medium Change

The step-wise reduction of sucrose concentration in the medium is one of the most widely used and effective ways to help tissues rehydrate and adapt from highly osmotic to a typical growth environment. In *Vitis*, rewarmed shoot tips were placed overnight on a culture medium with 0.6 M sucrose, followed by a medium with standard (3%) sucrose concentration [49]. Cassava shoot tips cryopreserved by programmed freezing were cultured on a series of solid media with sucrose concentration progressively decreasing every two days from 0.75 to 0.35 and then to 0.058 M [50]. In the later developed droplet-vitrification method, the recovery of cassava shoot tips was modified to 0.3 M sucrose for two days, followed by 0.058 M for one month [51]. The daily change of sucrose concentrations (0.5 M, to 0.3 M, to 0.1 M) was effectively used with shoot tips of chayote, *Sechium edule* [52]. Shoot tips of *Allium cepa, Oxalis tuberosa, Ullucus tuberosus*, and a large diversity of *Musa* species were recovered on a medium with 0.3 M sucrose for the first two days after rewarming [53,54,55]. One-day exposure to 0.3 M sucrose worked well for shoot tips of some cultivars of *Solanum tuberosum, S. commersonii, S. ajanhuiri,* and *S. juzepcukii* [56,57], shoot tips of *Araucaia angustifolia* [58] and embryogenic cell culture of cassava [59]. In the initial protocol for cryopreservation of potato collection at the International Potato Center (CIP), rewarmed shoot tips were transferred daily to a fresh medium with decreasing sucrose concentration (0.3-0.2-0.1 M sucrose) followed by recovery on medium with 0.07 M sucrose [60]. Later, this procedure was replaced by a 3-day maintenance of the rewarmed shoot tips on each of the prior mentioned sucrose concentrations; for the first nine days samples were kept in darkness [61,62]. The current potato cryopreservation protocol employed at CIP includes recovery for nine days on solid medium with 0.07 M sucrose (in darkness) [63]. This small change in the recovery process had a significant impact, improving the average survival and recovery rates to 77.4% and 71.5%, respectively compared to 69.7% and 59.5% achieved through step-wise recovery with reduced sucrose concentrations [63]. 

Taro shoot tips were first recovered overnight on a medium with 0.3 M sucrose before being transferred to 0.1 M sucrose-containing medium [64]. In relatively large and hard-structured explants, such as garlic shoot tips, the concentration of penetrating CPA in the tissues may remain high even after the unloading step, i.e., the explants may still be loaded with CPA when placed on a recovery medium [65]. It required one day of post-culture with 0.1 M sucrose for CPA to be entirely removed from the shoot tips and for sucrose concentration to reach the control level [65]. The concentration of cryoprotectants remaining in the shoot tips was negatively correlated with the sucrose concentration in the unloading solution and the post-culture medium [65]. It is worth noting that step-wise recovery on a sucrose-enriched medium may impose additional osmotic stress on plant tissues. Therefore, this strategy is unsuitable or should be carefully considered for osmotic-sensitive types of materials such as isolated root tips and shoot apices of tropical or wetland species.

Plant materials can be transferred to a fresh medium of the same composition once or several times during the recovery process (e.g., 5 h, 24 h or 48 h after rewarming, depending on species) to facilitate the removal of CPA and avoid the accumulation of toxic chemicals releasing from dead cells. Such medium changes are common in cell cultures [66,67,68,69] but were also used for shoot tips, e.g., for garlic [70] and *Garcinia cowa* [71].

### 3.2. Physical State of the Recovery Medium

The physical state of the recovery medium (liquid, semi-solid, solid or combinatory) may also impact regrowth. For example, recovery medium was one of the optimization points during the cryopreservation protocol development for potato shoot tips [31]. In some early protocols, shoot tips, after being cryopreserved by DMSO-based droplet-freezing, were embedded in warm agarose beads with or without PGRs, and placed on Petri plates. Once the drops solidified, a liquid nutrient medium was added [72]. Although these experiments were quite successful and resulted in 80% average survival and 40% regeneration for 219 accessions [73], later studies demonstrated the benefits of regeneration on solid medium (increased regeneration percentage and less callus formation [74]). When cryopreserved using a vitrification method, potato shoot tips recovered on solid medium showed over double the regeneration rate compared to liquid medium (58% vs. 21%) [75]. As a result, current cryopreservation protocols for potatoes at CIP and The Leibniz Institute of Plant Genetics and Crop Plant Research (IPK) employ recovery on solid medium only [48,63]. Diengdoh et al. [76] obtained different results when studying the interaction between the physical state of the medium (semi-solid-to-semi-solid or liquid-to-semi-solid) and its mineral composition for the recovery of *Paphiopedilum insigne* orchid protocorms. Recovery on liquid half-strength Murashige and Skoog (MS) medium for the first ten days, followed by semi-solid medium, resulted in the highest regrowth (37%) and shoot length (~3.7 cm) after using both vitrification and encapsulation-vitrification cryopreservation protocols.

Interestingly, undifferentiated plant cell cultures that normally exist as individual cells or cell aggregates suspended in a liquid medium usually benefit from the recovery on solid or semi-solid medium immediately after cryopreservation [26,68,77,78,79]. Alternatively, cells can be recovered on filter paper placed on a liquid medium [66]. Callus developing from the surviving cell clumps is then transferred to a liquid medium to re-initiate the suspension culture [80]. However, too early transfer of recovering cells from agar to liquid medium sometimes results in total loss of viability [68]. For example, Mikuła [68] noted that cryopreserved embryogenic cell cultures of *Gentiana tibetica* required at least three weeks on an agar medium for normal regrowth. Cell suspension of *Arabidopsis thaliana* could be successfully re-initiated after cell recovery for ten days on a semi-solid medium [81]. Cell culture of *Polyscias filicifolia* was recovered for one month as callus before cell suspension was re-established and used for the large-scale bioreactor cultivation [82]. Likewise, in *Dioscorea deltoidea* and *Panax ginseng*, a 30–40-day-long callus stage was reported before the re-initiation of the cell suspensions [77]. The main advantage of the recovery through callus on the solid medium is likely due to an avoidance of severe osmotic shock caused by quick rehydration of the plasmolyzed cells. Another consideration outlined by Nausch and Buyel [25] is that in case of low survival, cell densities above a certain threshold are necessary to initiate and sustain growth, which can be achieved by clustering cells on a solid medium. By contrast, *Medicago sativa* cell culture cryopreserved for 27 years formed proliferating cell suspension upon direct transfer from cryo-tubes to liquid medium without intermediate callus formation [83]. It is likely that, in this case, 7% DMSO used for cryoprotection did not cause severe osmotic stress and could be removed rapidly from the cells.

In conclusion, recovery on a solid or semi-solid nutrient medium was beneficial in most of the comparative studies. It can be recommended for all plant materials, including those typically cultured in the liquid phase, such as cell suspensions.

## 4. Strategy 2. Chemical Environment: Modifications of Recovery Medium Composition

Manipulating the mineral salt composition of the recovery medium is an effective strategy to improve the survival and regrowth of some species after cryopreservation. This strategy most often implies the modulation of iron and ammonium ion concentrations, but the effects of the carbohydrate source and gelling agent have also been tested.

### 4.1. Iron-Free Recovery Medium

The potential positive effect of removing Fe cation from the recovery medium was put forward by Benson et al. [84]. The authors outlined that transition metals, particularly iron, play a significant role in ROS-mediated oxidative stress. Referring to the Fenton chemistry (reduction of Fe^2+^ by hydrogen peroxide (H_2_O_2_) resulting in the formation of hydroxyl radical, OH^•^) and Haber–Weiss reaction (reduction of Fe^3+^ by superoxide and the further production of toxic hydroxyl radicals from H_2_O_2_), the authors suggested that the presence of Fe cations in the recovery environment may aggravate the oxidative stress occurring in plant tissues during the main steps of and immediately after cryopreservation (see below). Since H_2_O_2_ and superoxide radicals are usually elevated in plant tissues following cryopreservation, their co-presence with Fe cations may drastically increase ROS production. However, in the authors’ work, removing cations and Fe-EDTA from the recovery medium did not statistically affect the regrowth of rice cells [84]. Adding the Fe^3+-^chelating compound Desferrioxamine (0.5–10 mg L^−1^) to the recovery medium improved the regrowth of the two rice cell lines tested, only when coupled with cation-free medium (lacking main inorganic salts that can eventually produce cations). The authors suggested that this positive effect might be attributed to binding trace amounts of Fe or Cu in the tissues, thus reducing oxidative stress development [84]. Although the results did not support the straightforward conclusions, this study has drawn attention to the role of Fe source in the recovery process. Experimenting with ascorbic acid (AsA), a powerful antioxidant compound, Uchendu et al. [85] noted that its highly positive effect on the recovery of cryopreserved blackberry shoot tips could be revealed only in combination with an Fe-free medium. Since then, iron sources, particularly Fe-EDTA, have been generally excluded from the medium enriched with AsA for better regrowth [86,87]. Carmona-Martín et al. [88] used a medium with iron salt of ethylenediamine-N,N’-bis(2-hydroxyphenylacetic acid) (Fe-EDDHA, 6% Fe) (85.7 mg L^−1^) instead of Fe-EDTA (12% Fe) to support the regrowth of *Asparagus officinalis* rhizome buds after cryopreservation. Fe-EDDHA is a potentially attractive alternative to Fe-EDTA and was proven to be a preferable iron source for in vitro plant propagation of the date palm (*Phoenix dactylifera)* [89] and peach rootstock in vitro rooting [90]. At optimum concentration (93.5 mg L^−1^), Fe-EDDHA resulted in the highest callus growth, shoot regeneration, and the number of shoots, and decreased antioxidant enzymes catalase (CAT) and peroxidase (POD) activities compared to Fe-EDTA [89].

### 4.2. Ammonium-Free Recovery Medium

Ammonium salts are a standard component of in vitro culture medium, but they may be harmful to plant materials experiencing severe stress [91] and can be removed or reduced in quantity for better post-LN regrowth. Even low concentrations of ammonium nitrate may inhibit the normal recovery of shoot tips and undifferentiated cell cultures Table 1 [92,93,94,95]. Removing or substituting ammonium in the regrowth medium improved the regeneration of the cryopreserved shoot tips of *Holostemma annulare*, *Ipomoea batatas*, *Chrysanthemum morifolium* var. ‘Borami’, *Citrus limon* var. ‘Frost Eureca limon’ and *Aster altaicus* var. *uchiyamae* [93,94,95,96,97,98]. Regeneration improvement compared to the ammonium-containing medium varied from 17 to over 60% depending on species/plant material; 30–40% improvement was the most common (Table 1). For example, an ammonium-free medium improved the recovery of cryopreserved sweet potato shoot tips from 32% to 93% [97]. Alternatively, Jitsopakul et al. [99] reported better survival and development of cryopreserved *Bletilla striata* protocorms on ammonium-containing regrowth medium compared to the ammonium-free variant (66% vs. 32%). No effect of ammonium was recorded during the regrowth of the cryopreserved shoot tips of *Dioscorea alata* [100].

Data regarding the application of ammonium-free medium during the pre-LN steps of the cryopreservation protocol are controversial. As shown for shoot tips of silver birch, applying an ammonium-free medium may be beneficial during preconditioning, preculture, and cryoprotection treatments [91]. In *Pogostemon yatabeanus*, removing ammonium from the medium used at preculture, cryoprotection, and unloading steps significantly reduced regeneration regardless of the ammonium presence in the regrowth medium [107].

In most studies, the ammonium-free medium was the most effective when used immediately after rewarming. The duration of explant culture without ammonium was also critical. Too short exposure produces little or no benefit, while too long exposure suppresses the normal development of plantlets or cell division. With *Pogostemon yatabeanus* shoot tips, 1–3 days of regrowth on the ammonium-free medium was less effective than 5 days [107]. Excluding ammonium during the initial 1, 3, or 7 days after cryopreservation was equally beneficial for the viability of cryopreserved *Lavandula vera* and *Oryza sativa* cells [92,101].

Nitrate (NO_3_^–^) is known to alleviate some toxic effects of ammonium [110]. However, in cryopreservation, substituting ammonium ions with nitrates showed controversial results. In *Betula pendula* shoot tips cryopreserved using the slow-freezing method, 53–58% of recovery was achieved when KNO_3_ substituted ammonium nitrate during the cold hardening, cryoprotection, unloading, and regrowth steps [103]. In *Pogostemon yatabeanus* shoot tips, excluding ammonium from the medium at cryoprotection and regrowth stages was more beneficial than its substituting or co-presence with NaNO_3_ [107]. These data suggest that the toxic effect of ammonium during cryopreservation is tissue and genus-specific.

Interesting results were obtained when ammonium-free recovery was studied in relation to PGR content in recovery medium, light/dark conditions, and optimization of the cryopreservation protocol [107]. In this study, shoot tips of *Pogostemon yatabeanus*, an endangered species from the Korean wetlands known for its high sensitivity to dehydration and moderate susceptibility to toxic effects of CPA, were cryopreserved using the droplet-vitrification method. The harmful impact of ammonium during the first days of regrowth was most profound when a non-optimum CPA treatment was used at the pre-LN stage [107]. In this case, the ammonium-free medium significantly improved regrowth. However, if the damage caused by the cryopreservation process was too severe, e.g., in the treatments without preculture or with too long exposure to concentrated CPA, the shoot tips failed to recover even in the ammonium-free medium. The light/dark conditions showed no correlation with ammonium presence in the recovery medium. The authors concluded that a three-step regrowth procedure starting with (1) ammonium-free medium with 1 mg L^−1^ gibberellic acid (GA_3_) + 1 mg L^−1^ 6-benzylaminopurine (BA), followed by (2) ammonium-containing medium with the same PGRs, and (3) ammonium-containing medium without PGRs was essential for the development of healthy plants from cryopreserved shoot tips. The most impactful conditions for normal regeneration of both cryoprotected and cryopreserved shoot tips were as follows: PGRs at step 2 > ammonium-free medium at step 1 > PGRs at step 1 > PGR-free medium at step 3 [107].

The mechanism of ammonium toxicity during and after cryopreservation remains unclear. In plants, ammonium may cause the deregulation of pH homeostasis, ion imbalance, impaired nitrate signaling, disruption of hormonal homeostasis, and oxidative stress [111,112]. We might speculate that ammonium aggravates the oxidative stress in plant tissues during the cryopreservation process. Cryopreservation stress may reduce the metabolic activity of the explants, and the key enzymes of ammonia nitrogen metabolism could be inactivated or retarded several days after rewarming, leading to the accumulation of toxic levels of ammonium [91]. Hence, omitting ammonium in the regrowth medium for the first 5–7 days may benefit recovery and is worth testing when non-optimized protocols are used.

### 4.3. Carbohydrate Source and Gelling Agent in Recovery Medium

There are few reports on the comparative testing of different carbohydrate sources during the regrowth stage. As a universal energy source, sucrose has been used in most of the successful cryopreservation reports [19,24,30,31]. However, for shoot tips of strawberries, glucose produced higher regeneration when used throughout the cryopreservation process, including the regrowth stage [113]. The developed method using glucose instead of sucrose was successfully implemented for cryopreserving a collection of 28 *Fragaria* cultivars [113].

Gelling agents may affect the physiochemical characteristics of the culture medium through differences in elemental and organic impurities and the diffusion rate of nutrients [114,115]. Different gelling agents are available commercially, including agar, gellan gum (Gelrite, Phytagel, etc.), and agarose. As a gelling agent, gellan gum has a lower mineral content, fewer impurities of organic substances [116,117], better water availability [118], and better facilitates the diffusion of inhibitive molecules such as phenols [119], compared to agar. Thus, it has advantageous ability to stimulate the growth and development of in vitro plants during micropropagation and microtuberization [120], and stimulate shoot multiplication [121], germination [122], conversion of polyembryoids into plantlets [123], callus induction and roots development [115]. A possible reason for agar’s inferiority compared to gellan gum lies in the immobilization of up to 30% MS medium salts in the gel [124]. However, agar, Gelrite and Phytagel were all effectively used in the medium for post-cryopreservation recovery of different plant materials, including shoot and root tips, embryogenic cultures, somatic embryos, tubers, etc. [6,61,125,126,127,128,129,130]. The choice of the gelling agent likely depends on what is available for everyday use in the laboratory. Sometimes, the gelling agents were used in sequence. For example, cryopreserved somatic embryos of coriander were first recovered on a Gelrite-solidified medium, but agar was used for embryo maturation and plant development [127]. Several studies suggested that undifferentiated cell cultures generally yield better results when recovered on agarose [131]. Van Eck and Keen [69] compared medium solidified with 0.75% agarose or 0.8% agar for the post-cryogenic recovery of tobacco cell culture and found that cells recovered much faster on agarose-solidified medium and had approximately four times the cell mass compared to agar medium. It is also important to note that Gelrite may produce hyperhydricity, especially when combined with BA [132].

## 5. Strategy 3. Application of Exogenous Bioactive Compounds at the Recovery Stage

### 5.1. Cryopreservation and Oxidative Stresses

As discussed above, cryopreservation and subsequent rewarming are associated with extensive osmotic and temperature stress, toxic CPA action, potential mechanical damage, and other hazardous events resulting in substantial physiological changes in plant tissues [133]. It is therefore not surprising that cryopreservation provokes oxidative stress usually reflected by the formation and accumulation of ROS, such as OH^•^, H_2_O_2_, superoxide anion radical (O_2_^•¯^) and singlet oxygen (^1^O_2_) [134], as well as malondialdehyde (MDA), a product of membrane lipid peroxidation. Starting from the 1990s, cryopreservation studies and reviews increasingly emphasized the role of cryopreservation-induced oxidative stress as one of the major factors hampering post-rewarming survival and regeneration [135,136,137,138,139]. Expressions of oxidative stress-associated genes and a substantial shift in protein metabolism during and after cryopreservation has been well documented [140,141,142,143]. Recently, evidence has been built that ROS-triggered programmed cell death (PCD) may also happen during cryopreservation [144,145,146].

The most comprehensive review of the oxidative stress and antioxidant use during cryopreservation has been recently compiled by Ren et al. [146]. However, the number of detailed experimental studies on this topic is still limited. Elevated concentrations of ROS have been detected during all steps of the cryopreservation process [87,142,143,145,147]. With the droplet-vitrification or vitrification cryopreservation procedures, H_2_O_2_ accumulation was maximized during the dehydration and rewarming or unloading steps, depending on the type of the material [141,148,149,150]. Singlet oxygen production usually peaked after rewarming, and the most elevated concentrations of OH^•^ were detected in tissues after treatment with concentrated CPA and through the unloading and recovery steps [141,146,148]. MDA level was overall very high throughout the cryopreservation process and significantly upscaled during the CPA treatment [85,87,146,151]. The preliminary conclusion that can be drawn from the few published studies is that treatment with highly concentrated CPA and the period immediately after rewarming and washing off the CPA is associated with a burst in ROS formation. Therefore, alleviation of ROS during the first hours and perhaps days after rewarming is critical for the survival and regrowth of the cryopreserved materials.

It is now evident that the inability to cope with oxidative stress may be the major constraint for the successful recovery of species or genotypes cryopreserved through the non-optimized protocols. For example, the MDA level in cryopreserved *Passiflora ligularis* embryos during the recovery process negatively correlated with the time of CPA (vitrification solution) exposure during the pre-LN stage, having been the lowest for the treatment with the highest recovery (60 min) [152]. Meanwhile, the activities of enzymatic antioxidants CAT, superoxide dismutase (SOD), and AsA peroxidase (APX) were at their highest after optimum CPA exposure. Funnekotter et al. [153] found a strong negative correlation between the endogenous content of AsA in the shoot tips of three native Australian species and their post-cryogenic survival. In the same study, the half-cell reduction potential in the recovering shoot tips showed a solid correlation with the level of endogenous glutathione. The optimized cryopreservation protocol is likely to produce lower ROS throughout the process, giving the plant tissue a better chance to cope with the elevating oxidative stress.

The antioxidative systems in plant tissues may be severely damaged during cryopreservation, and therefore the application of exogenous antioxidants is expected to benefit survival and regrowth. This is particularly important for wild species where non-optimized procedures, “inherited” from other plants, e.g., crops, are often used with minimal tailoring.

### 5.2. Application of Compounds with Antioxidant Activity at the Recovery Stage

Among exogenous ROS scavengers, AsA remains the most widely used [87,154,155], Table 2. Both AsA and vitamin E (a water soluble mixture containing tocopherols, tocotrienols, and α-tocopheryl polyethylene glycol 1000 succinate) added at the pretreatment, osmoprotection, unloading, and regrowth steps of the cryopreservation procedure were effective in improving regrowth and reducing lipid peroxidation in *Rubus* shoot tips [85]. Importantly, AsA required an iron-free medium to reveal its positive effect at the regrowth step. Regrowth with AsA alone was not significantly different from the combined application of vitamin E + AsA. Vitamin E applied independently at the recovery step produced significantly less regrowth than its combination with AsA. However, the regrowth percentage was still considerably better than the control without vitamins. This difference in the activities of the two vitamins may be due to their different modes of action. While AsA may act as a direct ROS scavenger, the lipophilic α-tocopherol may be embedded into membranes and preferentially oxidized during the free radical attack as compared to polyunsaturated fatty acids. An ascorbate cycling mechanism reduces the resulting quinone to regenerate α-tocopherol [136]. However, for *Paphiopedilum insigne* protocorms, AsA and tocopherol produced little or no positive effect. They were less effective than glutathione and phloroglucinol in the same concentration range of 10–50 µM [76].

Glutathione is a low–molecular weight tripeptide sulfur compound that contains the thiol group (S–H) in the cysteine component (GSH). It protects oxygen-sensitive enzymes and proteins from the oxidative degradation of their sulfhydryl groups [136]. Glutathione at 0.03~0.16 mM added at preculture, CPA treatment, or recovery step provided 20–35% regrowth improvement in diverse cryopreserved materials (*Agapanthus praecox* callus, *Arabidopsis thaliana* seedlings, and *Paphiopedilum insigne* protocorms [76,149,154]). The use of GSH at preculture and CPA treatment steps (0.13 mM) and during the recovery after cryopreservation (0.0325 mM) also improved the survival of shoot tips of commercial *Citrus* varieties, and the optimized protocol was used to cryopreserve 13 cultivars [158].

Lipoic acid (2–6 mM), reduced glutathione (0.08–0.33 mM), and glycine betaine (10 mM) applied at the post-LN step improved the regrowth of *Rubus* shoot tips by ∼10–25%, while polyvinylpyrrolidone (PVP, 1–10 mM) had a negative or neutral effect [157]. Low concentrations of AgNO_3_ (5 µM) or PVP (0.25%) improved shoot regrowth from *Arachis hypogaea*, and two wild *Arachis* species shoot tips [164]. The use of exogenous CAT or malate dehydrogenase (MDH) (200–400 U/mL) immediately after cryopreservation led to a 23% and 41% increase in the survival of *Magnolia* and *Paeonia* pollen, respectively, with 400 U mL^−1^ giving the most significant improvement [165,166]. Phloroglucinol added at both the preconditioning and regrowth steps improved the regrowth of *Paphiopedilum insigne* protocorms [76]. Yet, phloroglucinol seemed to be more efficient when added to the CPA solution during the cryoprotection/dehydration stage [167]. As mentioned, desferrioxamine, an iron-chelating drug that reduced free radicals in animal tissues exposed to low temperatures, positively affected the post-thaw recovery of rice cells when combined with a cation-free medium [84].

Melatonin (0.1 and 0.5 µM) added to the preculture step of the cryopreservation protocol and recovery medium notably improved the regeneration of American elm (*Ulmus americana*) shoot tips cryopreserved using vitrification and encapsulation-vitrification methods [129]. The application of this compound at both steps produced higher recovery than its use at either the preculture or regrowth step. The inclusion of melatonin into alginate beads for cryopreservation and the subsequent regrowth of yam shoot tips (*Dioscorea alata* and *D. cayenensis*) improved plant regeneration from 5–15% to 10–35% [159]. The positive action of melatonin during cryopreservation is likely due to its activity as a direct ROS scavenger and as a phytohormone playing a vital role in the signaling networks and metabolic regulation [168]. Whelehan et al. [40] also pointed out that some metabolites formed when melatonin reacts with ROS are highly effective free radical scavengers [169,170]. For example, cyclic-3-hydroxymelatonin has been reported to prevent ROS damage, specifically to cytochrome c, and induce the cascade of effects for preventing programmed cell death [40,170].

In conclusion, oxidative stress imposed at all steps of the cryopreservation procedure may harm explant recovery, hampering the broader application of plant cryopreservation. The inability to cope with the escalating oxidation may underly the differences between the cryopreservation-sensitive and cryopreservation-tolerant materials or genotypes. Plant materials sensitive to CPA’s osmotic and chemical toxicity (e.g., species originating from wetlands or tropics) and wild species cryopreserved via procedures that are not fully optimized may particularly suffer from oxidative stress. Exogenous antioxidant application may result in 20–35% regrowth improvement after cryopreservation, depending on the protocol and material type, and are worth testing when cryopreserving sensitive materials. The past decades have provided new insights into the oxidative stress mechanisms and the function of the antioxidant system under cryopreservation. However, there is still a lot to explore on this topic. For example, it is often unclear how effective the antioxidant compounds are if supplied solely at the recovery stage.

### 5.3. Application of Polymeric Compounds, Nonoparticles, and Antimicrobial Agents at the Recovery Stage

Several studies explored the potential benefits of ice-blocking agents added at different steps of the cryopreservation protocol and in the medium for regeneration. One of these compounds was Supercool X1000, a partially hydrolyzed polymer of polyvinyl alcohol with a function similar to antifreeze proteins [161]. When added to a CPA solution PVS2 at the dehydration step, Supercool X1000 at concentration 0.1% or 1.0% led to increased regrowth (by 26–30% on average) of cryopreserved seeds of *Oncidium flexuosum* orchid and shoot tips of two potato cultivars [161,167]. However, the ice-blocking activity of such compounds makes them most effective at cryoprotection and in the freezing-rewarming steps rather than during the recovery treatments.

Polyoxyethylene (POE)-polyoxy propylene (trade name Pluronic F-68, Gibco^TM^) added to the culture medium (0.005%) after cryopreservation enhanced regeneration of shoot tips in two potato varieties; 76% and 43% versus 43% and 27%, respectively [161]. In *Oryza sativa* cv. Taipei 309 cell culture, the addition of Pluronic F-68 to the regrowth medium led to a 36% viability increase in cryopreserved cells compared to cells recovered on the usual medium [162]. Supplementing the regrowth medium with oxygenated perfluorocarbon resulted in a 20–24% improvement in cell viability following cryopreservation. The combination of this substance with Pluronic F-68 resulted in the most pronounced (up to 57%) viability increase over the control [162]. Pluronic F-68 was reported to act as a cryoprotective agent in animal cells and is believed to protect cells against fluid-mechanical damage and even promote cell division [162]. The combination of oxygenated perfluorodecalin and medium supplemented with Pluronic F-68 (0.01%) increased the mean plating efficiency of protoplasts by 52% above control, thus promoting cell proliferation [171]. Oxygenated perfluorodecalin is an inert organic compound that can dissolve substantial volumes of respiratory gases and has been used for enhancing oxygen transfer rates in different organisms cultured at the interface of liquid and solid phases [171]. The mechanism of its positive effect during regrowth after cryopreservation is poorly understood, but it might involve increasing gas exchange or the absorption of growth-suppressing compounds.

Activated charcoal is thought to absorb toxic substances, mainly phenolic compounds, that injured plant tissues may release to the environment. As such, it is sometimes added by default to the recovery medium [24,130,172]. However, the assumption of its beneficial effect is not always correct. The presence of activated charcoal reduced the regrowth of cryopreserved shoot tips of *Arachis hypogaea* [164] and grape cells [173]. It was not beneficial for the regrowth of *Pogostemon yatabeanus* shoot tips [107]. On the other hand, activated charcoal (2 g L^−1^) added to the recovery medium notably improved the regeneration of *Dendrobium* Sonia-28 after cryopreservation [156]. However, this positive effect could be due to the presence of AsA in the recovery medium during the same experiment.

More recent studies also explored the application of nanoparticles (NP) of different origins for plant cryopreservation, with gold and carbon nanomaterials being potentially promising [30,146]. Kulus and Tymoszuk [163] reported that 10 ppm of gold nanoparticles (AuNPs) added into the protective alginate bead matrix for cryopreservation of *Lamprocapnos spectabilis* shoot tips improved their recovery level by ca. 20%. On the other hand, the presence of nanoparticles in the recovery medium had a deleterious effect on shoot tip survival. Adding single-wall carbon nanotubes or fullerene (C60) at the pre-LN steps during cryopreservation of *Agapanthus praecox* cell culture reduced ROS accumulation [148,174]. It enhanced the activity of the antioxidant enzymatic machinery in tissues leading to improved survival [148,174]. However, the effect of most NP at the recovery stage was not yet tested.

Some in vitro stock cultures may contain endophytes that remain invisible during subcultures but become evident after the tissues experience the stress of a cryopreservation procedure. Moreover, the purposeful filling of cryovials with LN (a common practice in some cryopreservation methods) may have consequences in the form of cross-contamination by microbial agents and pathogens [28]. In these cases, antibiotics or antimicrobial compounds can be added to the recovery medium to suppress the growth of contaminating organisms or clean up the culture [14]. For example, in vitro rhizome buds of *Asparagus officinalis* were sterilized for the second time after cryopreservation and recovered on medium with the antibiotic Cefotaxime (200 mg L^−1^) to reduce bacterial contamination [88]. Plant Preservative Mixture (PPM, 1 mL L^−1^), a commercial anti-microbial product (Plant Cell Technology, USA), was added to the medium for the recovery of cryopreserved potato shoot tips to avoid potential contamination [175].

## 6. Strategy 4. Plant Growth Regulators and Their Combinations at the Post-LN Stage: Modulation of the Physiological Response

Plant growth regulators are essential for the recovery and regrowth of cryopreserved plant tissues. Depending on the PGR balance, the recovery process may be shifted towards cell proliferation, morphogenesis, or embryogenesis. It is not uncommon when the recovery process is done through two or more steps, each implying different composition of PGRs for a more precise control of the morphological response in the cryopreserved tissues. During the first days after rewarming, PGRs must support the process of reparation, survival of cell clusters in the injured tissues, and their proliferation. At later stages, the type of morphogenic response becomes most important. It has been generally accepted that in vitro conditions should be thoroughly optimized before cryopreservation and can be mimicked during the recovery process [33]. However, this assumption is not always accurate since some species showed specific requirements for medium composition and PGRs immediately after cryopreservation [31,49,107,176,177,178].

Moreover, there is a growing challenge of cryopreserving endangered species without an established efficient/optimized in vitro protocol [2,21]. Another challenge is linked to the large-scale application of cryobanking to national and world collections of plant genetic resources. In these cases, a single protocol with few modifications should be applied across a wide range (over 1000) of genotypes and ensure recovery above 40%, a threshold line accepted in most genebanks [9,51,61]. Knowledge of the general requirements for medium composition, particularly PGRs for different types of propagules, may help quickly establish an operating cryopreservation procedure with satisfactory recovery. Regrettably, only a few studies explored the effect of PGRs in cryopreservation procedures and provided a comparative analysis of their action [107,179,180].

To better understand the patterns of PGRs applied during the recovery process, we analyzed publications on the cryopreservation of different types of plant materials, including shoot tips, undifferentiated cell cultures (callus or suspension), embryogenic cultures, somatic embryos, alternative propagules (tubers, rhizomes, protocorms, adventitious buds), hairy roots, and adventitious root cultures. No less than twenty-five research papers with publication dates between 1984 and 2022 were analyzed for each material type. At least five representative studies for each material type are included in Table 3, Table 4, Table 5 and Table 6. These studies represent different cryopreservation protocols (programmed/slow freezing, droplet-vitrification, vitrification, encapsulation-dehydration, preculture-desiccation, etc.) and mineral salt compositions used at the recovery stage. Older research papers published in the 1980s to the 1990s were examined to track the evolution of PGR composition during the recovery stage as a result of protocol optimization and new protocol developments, if any. Our findings confirmed that the use of PGRs is often specific to the type of material and evolved in line with the optimization of the cryopreservation procedures, as discussed below.

### 6.1. Plant Growth Regulators for Shoot Tips and Axillary Buds

In the cryopreservation of differentiated, organized tissues such as shoot tips, axillary buds, etc., the major issue to be avoided is callus formation bearing a potential risk of genetic variation in regenerants [14,33]. Hence, PGRs that induce dedifferentiation, particularly 2,4-dichlorophenoxyacetic acid (2,4-D), should be minimized [33]. Thidiazuron (TDZ) is another PGR to avoid immediately after cryopreservation due to reported hyperhydricity and abnormal shoot development in some species in vitro [21]. Among the PGRs, BA and GA_3_ applied solely or combined with low levels of auxins usually produce good results, promoting direct plant formation from cryopreserved shoot tips. In a recent comprehensive review, Normah et al. [24] compiled detailed cryopreservation protocols applicable to the shoot tips of tropical plant species, including regrowth conditions. In 10 out of the 25 plant species reviewed, BA was used as the only PGR at the initial recovery step, with the concentration varying from 0.05 to 4 mg L^−1^. Combinations of BA with GA_3_ (up to 1 mg L^−1^) were used in 20% of the reviewed studies. In five species, BA was applied together with up to 0.5 mg L^−1^ auxins [indole-3-acetic acid (IAA), 1-naphthaleneacetic acid (NAA), or indole-3-butyric acid (IBA)], but the auxin concentration was at least twice or, more frequently, 10-fold lower than that of BA [33]. In agreement with these findings, GA_3_ and BA combinations with zeatin and IAA were the most common for recovering shoot tips of clonally propagated crops [30]. Some of the standard recovery protocols developed for agriculturally important plants in world genebanks and applied through a range of genotypes are listed in Table 3.

**Table 3:** Examples of plant growth regulators used at the recovery step during cryopreservation of shoot tips of forest and fruit trees and agronomically important species in world genebanks.

Species	Explant	Cryopreservation Method	Regrowth Medium	Plant Growth Regulators (mg L^−1^)	Survival (S) or Regrowth (R) Response	Reference
**Shoot tips of agronomically important species (protocol implementation to multiple accessions)**						
*Allium* spp.	Shoot tips	DV	B5	2-iP 0.5 + NAA 0.1 → no PGR	R: >40% for 12 accessions	[70]
*Allium* spp.	Clove apices, bulbil primordia	DV	MS	IAA 0.3 + 2-iP 2.0	R: 65.9% (mean of 1158 accessions)	[6]
*Musa* spp. + 1 *Ensete* spp.	Shoot tips, meristematic clumps	DV	MS	BA 0.2	R: 52.9% for shoot tips (average of 56 accessions)	[55,181]
*Solanum* spp.	Shoot tips	DV	MS	GA_3_ 0.1 + kinetin 0.4 + coconut water 20 mL L^−1^	S: 68.8% R: 55.4% (mean of 1028 accessions)	[7]
*Solanum tuberosum*	Shoot tips	DMSO-droplet freezing	MS	zeatin 0.5 + GA_3_ 0.2 + IAA 0.5	R: 54% (mean of 28 accessions)	[48]
		DV			R: 71% (mean of 28 accessions)	
*Manihot esculenta*	Shoot tips	DV	MS	kinetin 0.5 + GA_3_ 0.25	R: 0–100% (97 accessions)	[51]
*Vitis* spp.	Shoot tips	DV	1/2MS (macro)	BA 0.2	R: >43% for 13 genotypes	[49]
*Fragaria* spp.	Shoot tips	Vitrif	MS	BA 0.1 + GA_3_ 0.01 + IAA 1 + adenine sulfate 80	R: 75–100% (194 genotypes)	[182]
**Woody species**						
*Betula lenta*	Shoot tips	DV	DKW	BA 0.1 + GA_3_ 0.35	R: 52%	[183]
*Ulmus americana*	Shoot tips	Vitrif Enc-Vitr	DKW	BA 0.5 + GA_3_ 0.1	R: 50–63%	[129]
*Pyrus* spp.	Shoot tips	Vitrif	MS	BA 0.1 + NAA 0.01 + GA_3_ 0.1 or BA 1 only	R: 33% (average of 22 accessions)	[17]
*Ulmus minor, U. laevis, U. glabra*	Dormant buds	SF	MS	BA 0.1	R: 42–76	[126]
*Malus* spp. (4 species, 9 genotypes)	Shoot tips	Enc-Deh	MS	BA 0.25 + IBA 0.01	R: 57% (mean of 9 genotypes)	[184]
*Malus x domestica* (4 genotypes)	Shoot tips	Enc-Deh Enc-Vitr	MS	BA 0.5 + IBA 0.05	R: 65–88%	[176]
*Populus tremula x Populus tremuloides*	In vivo buds	SF	WPM	BA 0.5 + IAA 0.5	R: 72–96%	[185]

SF—slow/programmed freezing; Vitrif—vitrification; Enc-Deh—encapsulation-dehydration; Enc-Vitr—encapsulation-vitrification; DV—droplet-vitrification. MS—Murashige and Skoog medium; B5—Gamborg medium; DKW—Driver and Kuniyuki medium; WPM—Woody Plant Medium.

In cassava cryopreservation, the response after LN was affected by the type of cytokinin used and its concentration [50]. Kinetin at 0.5 mg L^−1^ was more efficient than 6-(γ,γ-dimethylallylamino)purine (2iP), TDZ, and adenine. Increasing BA concentration from 0.04 to 0.5 mg L^−1^ was also beneficial. Reducing NAA (to 0.01 mg L^−1^) and increasing GA_3_ (to 0.5 mg L^−1^) in the recovery medium diminished callus growth and stimulated the elongation of shoots [50]. With *Rubus* spp. shoot tips, IBA was a standard component of the multiplication medium, but it should be removed at the post-LN stage to reduce callus formation and allow for the direct regeneration of shoots from the meristematic region [186]. In the earlier reports on the cryopreservation of potato shoot tips, modifications of PGR content in the recovery medium led to improved shoot production [31,187]. The recovery medium initially contained a combination of 0.5 mg L^−1^ IAA + 1.0 mg L^−1^ GA_3_ + 0.5 mg L^−1^ zeatin [187]. The latter was then substituted by 0.04 mg L^−1^ kinetin, which increased the regeneration of shoots but still through callus formation [187]. Modified PGRs (IAA 0.5 + GA_3_ 0.2 + zeatin 0.5 mg L^−1^) in the agarose drops used for shoot tip post-cryopreservation recovery positively affected plant regeneration in ten potato cultivars compared to agarose drops without PGRs [31,188]. The evolution of the potato cryopreservation protocol at CIP also led to the adjustment of the recovery step [7,61]. In 2006–2012, the standard protocol employed GA_3_ 0.1 + kinetin 0.4 mg L^−1^ in the recovery medium. Since 2013, this medium was also enriched with 20 mL L^−1^ coconut water [7]. The recovery protocol for potatoes at IPK utilized GA_3_ 0.2 + zeatin riboside 0.5 + IAA 0.5 mg L^−1^ [48].

Likewise, for tree species cryopreserved using shoot tips of in vitro grown plants or bud meristems, BA alone and its combination with GA_3_ or low auxin concentrations were most effective (Table 3). Rathwell et al. [183] compared various combinations of GA_3_, BA, kinetin, and zeatin in a regrowth medium for *Betula lenta*. They found that GA_3_ (0.35 mg L^−1^) ensured the best plant regeneration from both cryoprotected and cryopreserved shoot tips compared to all other treatments. Shoot tips of *Ulmus americana* were successfully recovered in the presence of GA_3_ and BA [129], while BA alone supported regrowth in other *Ulmus* species [126]. A medium for the recovery of shoot tips from fruit trees and *Populus* also contained BA/GA_3_ and low levels of auxins (Table 3) [176,184,185]. Some researchers used high levels of PGRs, e.g., 10 mg L^−1^ zeatin, for shoot tips of olive [189], but such examples are rare.

In rare plant species *Anigozanthos viridis* ssp. *terraspectans*, a combination of cytokinin and 0.17 mg L^−1^ GA_3_ in recovery medium from day zero after rewarming was the most appropriate for obtaining vigorous plantlets from the cryopreserved shoot tips [179]. For *Pogostemon yatabeanus*, an endangered Korean species from wetlands, a combination of GA_3_ 1 + BA 1 mg L^−1^ was found to be the most suitable for regrowth after cryopreservation among various mixtures of GA_3_, BA, and zeatin. The lowest recovery was recorded on medium without PGRs [107].

Presumably, BA and GA_3_ applied alone or in combination with low auxin levels (IAA, NAA, IBA) may be the most suitable for shoot tip regrowth in a range of taxa, including crops, trees, and endangered species. These combinations might be recommended at the start of protocol development or initial protocol tests for species with non-optimized in vitro culture.

### 6.2. Plant Growth Regulators for Cell Cultures

In contrast to shoot tips, undifferentiated cell cultures needed auxins, preferably 2,4-D, to restore their proliferation ability after cryopreservation (Table 4). When a cytokinin, mostly kinetin, was used with 2,4-D or NAA, its concentration was usually ~10-fold lower compared to auxins.

**Table 4:** Examples of plant growth regulators used at the recovery step during cryopreservation of somatic embryos, embryogenic and non-embryogenic (undifferentiated) cell cultures.

Species	Explant	Cryopreservation Method	Regrowth Medium	Plant Growth Regulators (mg L^−1^)	Survival (S) or Regrowth (R) Response	Reference
**Somatic embryos**						
Several *Citrus* genotypes	SE	Enc-Deh	MS	No PGR	S: 76–100%	[12]
*Theobroma cacao*, 4 genotypes	SE	Enc-Deh	DKW	No PGR	R: 25–72% 33% plant conversion	[125]
*Olea europea*	SE	Enc-Deh Enc-Vitr	MS	No PGR	R: up to 54%	[190]
*Coriandrum sativum*	SE	Preculture-desic	MS	2,4-D 1.0	R: 98%	[127]
*Elaeis guineensis*	SE	Preculture-desic	MS	2,4-D 0.2	R: 80%	[191]
*Castanea sativa*	SE	Preculture-desic	MS	NAA 0.1 + BA 0.1	R: 68% (resumption of embryogenesis)	[192]
**Embryogenic/morphogenic cell cultures**						
*Quercus suber*	EC	Vitrif	SH	No PGR	88–93% embryo recovery, 60% plant regeneration	[193]
*Asparagus officinalis*	ES	Vitrif SF	LS	2,4-D 1.1	S: 82–86% Embryo production retained at the control level	[194]
*Arabidopsis thaliana*	ES	SF	MS	2,4-D 2	Regrowth through callus followed by shoot regeneration	[81]
*Kalopanax septemlobus*	EC	Vitrif DV	MS	2,4-D 0.1	R: 99% Embryo production retained at the control level	[195]
*Manihot esculenta*	EC	Preculture-desic	MS	BA 0.05 + NAA 0.01 (24 h) → BA 0.1	S: >90% Embryogenic competence: >90%	[59]
*Hevea brusiliensis*	EC	SF	MH	BA 0.3 + 2,4-D 0.3 + ABA 0.17	S: 40–70% depending on cell line Embryo production reported	[196]
*Pinus patula*	EC	SF	MS	BA 1.0 + 2,4-D 2.0	Good regrowth followed by embryo and plant development reported	[197]
*Pinus sylvestris*, several cell lines	EC	SF	DCR	BA 0.5 + 2,4-D 2 or 3	78% of cell lines remained viable and proliferated	[198]
**Undifferentiated cell cultures**						
*Triticum aestivum* cv. Norstar	Callus	SF	MS	No PGR	S: 82% R: 54.5% 18% calli formed plantlets	[199]
*Arabidopsis thaliana*	Susp	Enc-Deh	MS	NAA 11	S: 34% R: 100%	[78]
*Ginkgo biloba*	Callus	Preculture-desic	MS	NAA 5	R: 23%	[200]
*Panax ginseng*	Susp	SF	MS	2,4-D 1	Growth resumed	[201]
*Nicotiana tabacum* (transgenic lines)	Susp	Vitrif	MS	2,4-D 2.21	Regrowth through callus mentioned	[69]
*Bromus inermis*	Susp	SF	EM	2,4-D 0.05	R: 85%	[202]
*Catharanthus* *roseus*	Susp	SF	B5	2,4-D 1	S: 61.6%	[66]
	Susp	SF	B5	2,4-D 1 + kinetin 0.1	Growth resumed	[201]
*Polyscias filicifolia*	Susp	SF	MS	NAA 3 + kinetin 2	S: 40% Large-scale bioreactor cultivation resumed	[82]
*Medicago sativa*	Susp	SF	MS	2,4-D 1 + kinetin 0.1	S: 20%	[83]

SE—somatic embryos; EC—embryogenic callus; ES—embryogenic cell suspension; Susp—cell suspension. SF—slow/programmed freezing; Vitrif—vitrification; Enc-Deh—encapsulation-dehydration; Enc-Vitr—encapsulation-vitrification; DV—droplet-vitrification; desic—dessiccation. MS—Murashige and Skoog medium; DKW—Driver and Kuniyuki medium; B5—Gamborg medium; SH—Shenk and Hildebrand medium; LS—Linsmaier and Skoog medium; MH—Mueller–Hinton medium; DCR—Gupta and Durzan medium; EM—Erickson’s—medium.

Embryogenic cell cultures required a balance of auxins and cytokinins for recovery, and BA was most frequently used as a cytokinin (Table 4). Some of the cryopreserved embryogenic cultures could proliferate on a medium containing only auxin [81] or, in a few studies, without PGRs (Table 4). In the latter cases, the medium could be enriched with specific vitamins, glycine, antioxidants, or have a modified mineral composition for better regrowth. At later stages, the cultures could be transferred to a medium with ABA for embryo maturation.

Somatic embryos (Table 4) are probably the least demanding plant material regarding the medium composition. They could be recovered without PGRs or with low concentrations of auxins (up to 2 mg L^−1^).

### 6.3. Plant Growth Regulators for Root Explants

Successful cryopreservation of root explants isolated from in vitro grown plants and adventitious or hairy root cultures of several species has been reported [15,16]. Hairy and adventitious roots attract both scientific and commercial interest as an alternative source of plant-derived bioactive compounds with the potential for application in pharmaceuticals, cosmetics, and natural health products. Hairy roots result from plant tissue transformation by Ri (root-inducing) plasmid from *Agrobacterium rhizogenes* [203]. In contrast, adventitious roots are induced from non-transformed plant tissues in the presence of auxins and do not carry foreign genes [204]. Both hairy and adventitious roots readily proliferate in vitro with the possibility for large-scale cultivation in industrial bioreactors [205,206]. The cryopreservation of root tips and segments was achieved using the following methods: programmed freezing, droplet- and classical vitrification, encapsulation-vitrification, and encapsulation-dehydration, with regrowth of new roots ranging from 9–23% in earlier protocols to 70–100% in recent studies [16], Table 5. In most cases, successful recovery of hairy roots from LN does not require PGRs. Cryopreserved root cultures grew well and proliferated on a solid or liquid medium composed of basal salts and sucrose (Table 5). By contrast, a few studies on cryopreservation of adventitious root cultures suggest that they require auxins (NAA, IBA) in the regrowth medium for normal development and proliferation [207,208,209]. The cryopreservation of root segments excised from in vitro-grown plants was also tested as an alternative option. Such explants showed good survival and direct plant regeneration in the presence of cytokinins or GA_3_ [210,211,212]. Root tips and segment explants may serve as an excellent example of how visually similar plant materials require different types and combinations of PGRs during the post-LN treatment due to their initially different physiology and growth requirements.

**Table 5:** Examples of plant growth regulators used at the recovery step during cryopreservation of root tips and segments.

Species	Explant	Cryopreservation Method	Regrowth Medium	Plant Growth Regulators (mg L^−1^)	Survival (S) or Regrowth (R) Response	Reference
**Hairy roots**						
*Nicotiana rustica*	Root tips	SF	B5	No PGR	S: 83%; R: 23%	[213]
*Beta vulgaris*	Root tips	SF Ultra-Rapid	B5	No PGR	S: 42–46%; R: 6% S: 84%; R: 9.7%	[213]
*Maesa lanceolata* *Medicago truncatula*	Root tips	Enc-Deh	SH MS	No PGR	R: 90% R: 53%	[214]
**Adventitious roots**						
*Hyoscyamus niger*	Root tips	Vitrif	WPM	No PGR	R: 93%	[215]
*Panax ginseng*	Root tips	DV Vitrif	MS	IBA 5 + IBA 0.05	S: 90%; R: 32.5% R: 15%	[207,208]
*Tarenaya rosea*	Root tips	Enc-Vitr	MS	NAA 0.25	R: 91%	[209]
**Roots from in vitro grown plants**						
*Hypericum perforatum*, 5 lines	Root segments	DV	MS	GA_3_ 1.0	R: 45–78%	[212]
*Vanilla planifolia*	Root tips	DV	MS	BA or kinetin 1	S: 60%; R: 43%	[211]
*Passiflora pohlii*	Root tips	V-cryo-plate	Modified MS	IAA 1	R: 79%	[216]
*Cleome rosea*	Root segments	Vitrif	MS	NAA 0.25 for regrowth, BA 0.5 for shoot development	R: 100%	[210]

SF—slow/programmed freezing; Vitrif—vitrification; Enc-Deh—encapsulation-dehydration; Enc-Vitr—encapsulation-vitrification; DV—droplet-vitrification. B5—Gamborg medium, MS—Murashige and Skoog medium, SH—Shenk and Hildebrand medium, WPM—Woody Plant Medium

### 6.4. Plant Growth Regulators for Other Plant Materials

Microtubers, rhizome buds, and multiple adventitious buds are an alternative to shoot tips for the cryopreservation of some ornamentals and crops. One of the recent trends is the cryopreservation of adventitious buds regenerated from the stem of leaf discs on a culture medium [19]. This explant type was successfully tested with orchids, blueberries, shallots, and lilies [217,218,219,220,221]. Similar to in vitro-derived shoot tips, adventitious buds required moderate concentrations of PGRs and a balance of auxins and cytokinins to recover and produce shoots (Table 6). The use of adventitious buds possessed some crucial advantages over the conventional use of the shoot tips from in vitro grown plants, such as simplifying the whole process by eliminating the stage of shoot tip excision, ensuring higher shoot regrowth due to the high number of buds per explant, and easier manipulation [19]. However, Wang et al. [219] also pointed to the increased risk of genetic instability of the regenerants and the overly long cryopreservation cycle (18–19 weeks) versus 8–9 weeks for field-grown bulbs and 13–14 weeks for in vitro shoot tips.

**Table 6:** Examples of plant growth regulators used at the recovery step during cryopreservation of tubers, rhizomes, adventitious buds, protocorms, and protocorm-like bodies.

Species	Explant	Cryopreservation Method	Regrowth Medium	Plant Growth Regulators (mg L^−1^)	Survival (S) or Regrowth (R) Response	Reference
*Solanum tuberosum*	Microtubers	Preculture-desic	MS	No PGR	S: 100% R: 40–75% (shoot regrowth)	[130]
*Cymbidium kanran*	Rhizome sections	Preculture-desic	Hyponex	GA_3_ 1.0 + BA 0.5	R: 90%	[178]
*Asparagus officinalis*	Rhizome buds	Enc-Deh	MS modified with Fe-EDDHA	NAA 0.5 + kinetin 0.7	R: 84%	[88]
*Lilium* Oriental hybrid ‘Siberia’	Adventitious buds	DV	MS	NAA 1 + TDZ 0.2	S: 85% R: 72% (shoot regeneration)	[218]
*Vaccinium corymbosum*	Adventitious buds	DV	WPM	Zeatin 0.4	R: 100% (shoot regrowth)	[217]
*Allium cepa* var. *aggregatum*	Adventitious buds	DV	MS	NAA 0.1 + BA 0.5	R: 72% (shoot regrowth)	[219]
*Cattleya loddigesii* var. *hassisoniana*, *C. walkeriana*	Meristematic clusters (shoot primordia)	Preculture-desic	Hyponex	No PGR	R: 100%	[220]
*Dendrobium* cv. Yukidaruma	Meristematic clusters (shoot primordia)	Preculture-desic	Hyponex	No PGR	R: 100%	[220]
*Vanda pumila*	Meristematic clusters (shoot primordia)	Preculture-desic	B5	BA 0.02	S: 65%	[221]
*Dendrobium candidum* (*Dendrobium moniliforme*)	Protocorms	Vitrif	1/2MS	No PGR	R: 88%	[222]
*Grammatophyllum speciosum*	Protocorms	DV Enc-Deh Enc-Vitr	1/2MS	No PGR	R: 12–38%	[223]
*Oncidium hamana* “elfin”	Protocorm-like bodies (PLBs)	Enc-Deh	1/3MS	No PGR	R: 30%	[224]
*Caladenia latifolia*	Protocorms and PLBs	DV	1/2MS	Zeatin 0.2 + GA_3_ 0.17	R: 84–85%	[225]
*Cleisostoma areitinum*	Protocorms	Enc-Dowaeh	ND	BA 2 + NAA 2	R: 49%	[226]
*Phalaenopsis bellina*	Protocorm-like bodies (PLBs)	Enc-Deh	1/2MS	TDZ 3	R: 30%	[227]

PR—programmed freezing; Vitrif—vitrification; Enc-Deh—encapsulation-dehydration; Enc-Vitr—encapsulation-vitrification; DV—droplet-vitrification; desic—dessiccation. MS—Murashige and Skoog medium; WPM—Woody Plan Medium, B5—Gamborg medium, ND—New Dogashima medium

Orchid meristematic clusters and potato microtubers could recover on medium with traces or no PGRs in culture medium [130,220,221]. At the same time, the presence of kinetin, BA, and GA_3_ (up to 1 mg L^−1^) was needed for the regeneration of rhizome buds [88,178]. About 80% of all papers regarding the cryopreservation of orchid protocorms and protocorm-like bodies (PLBs) published before 2016 reported regrowth on the medium without PGRs [228,229]. Some authors added potato or banana extract to substitute PGRs in the recovery medium, which was quite common for orchid cultivation in vitro [105,226]. In the later reports, relatively high concentrations of auxins, cytokinins, GA_3_, or TDZ (up to 3 mg L^−1^) were used for the cryopreservation of some orchid species [225,227].

### 6.5. Unusual Plant Growth Regulators

Recently, some unusual growth regulators were also tested for their suitability in cryopreservation. An auxin-type compound, 2-naphthoxyacetic acid, was successfully used at a concentration of 1 mg L^−1^ to recover embryogenic cell suspensions of several *Vitis* cultivars, leading to survival ranging from 42 to 82% and subsequent plant regeneration [230]. Meta-Topolin [N6-(3-hydroxybenzyl)adenine], an aromatic cytokinin, was found to be effective during the recovery of *Corylus avellana* buds and resulted in the same or significantly better regrowth than BA, depending on cultivar [231]. Meta-Topolin is reported to increase in vitro shoot production, reduce abnormalities during development, and alleviate shoot tip necrosis in delayed senescence in different plant species [232]. In micropropagated *Spathiphyllum floribundum*, meta-Topolin, more effectively than other adenine-type cytokinins, supported both shoot production and root formation in vitro and during acclimatization [233]. Compared to BA, the compound also resulted in a higher content of total soluble proteins and amino acids in regenerants of *Daphne mezereum* produced from young shoot fragments in vitro [234]. Salicylic acid is known to be involved in oxidative stress responses and programmed cell death [40]. Applying salicylic acid at the pre-LN steps improved post-cryopreservation regrowth of a range of species and material types, such as axillary and apical buds of *Vitis* cultivars, axillary buds of *Solanum* buds, and embryonic axes of *Melia azedarach* [235,236,237]. However, its effect at the recovery step was not explored. Ancymidol (2 mg L^−1^), an inhibitor of gibberellin biosynthesis, was added to the culture medium to recover *Asparagus officinalis* rhizome buds after cryopreservation in combination with NAA 0.5 + kinetin 0.7 mg L^−1^ to support plant development from the survived explants [88].

### 6.6. Step-Wise Change of Plant Growth Regulators during the Recovery Process

While the regrowth of most propagules is usually performed in one stage, certain explants need two- or three-step recovery with varying PGR composition for the desired morphogenic response. For shoot tips and protocorms, a higher concentration of PGRs or a combination of GA_3_ with cytokinins or auxins may be used at an earlier recovery stage to stimulate cell recovery and proliferation, followed by medium with low GA_3_ or without PGRs for regeneration of normal plantlets. For example, shoot tips of *Kaempferia galaga* were recovered on medium with GA_3_ + BA but transferred to medium with BA + NAA for further regrowth [238]. Shoot tips of *Ipomea batatas* were recovered on medium with GA_3_ 1 + BA 0.5 mg L^−1^ for an initial seven days, but later BA was omitted, and GA_3_ was reduced to 0.5 mg L^−1^ [239]. Protocorms of the terrestrial orchid *Caladenia latifolia* were first recovered for one week on medium with zeatin and GA_3_, then moved to medium without PGRs but with coconut water for subsequent plant regeneration [225]. After the cryopreservation of osmotic stress-sensitive *Pogostemon yatabeanus* shoot tips, a 3-step sequential regrowth was necessary for the development of normal plants (91.6% regeneration), and the presence or absence of PGRs at each stage was vitally important [107]. The combination of GA_3_ 1 + BA 1 mg L^−1^ was beneficial but not critically important during the first five days after rewarming (recovery step 1), was most important at days 5–27 after cryopreservation (recovery step 2), and should be omitted for normal plant development after day 27 (at stage 3) [107]. Cryopreserved shoot tips of *Dioscorea alata* produced a maximum of 39% shoot regeneration when initially cultured for 40 days on medium with reduced ammonium concentration and a combination of BA 1.0 + zeatin 1.0 + IAA 0.15 + GA_3_ 0.2 mg L^−1^, followed by two sequential transfers to other media to promote normal shoot formation [100]. The step-wise change of PGR concentration to improve the plant regeneration of potato shoot tips in certain protocols was also highlighted by Kaczmarczyk et al. [31]. For example, some authors [240] used medium with IAA 0.05 + zeatin 0.3 + GA3 0.05 mg L^−1^ for one week followed by culture on medium without PGRs. In other protocols, GA_3_ in moderate concentrations was used solely at the latter stage of recovery [31,241].

We hypothesized that plant materials that are sensitive to the osmotic and chemical toxicity of CPA might benefit from the step-wise regrowth starting with ammonium-free medium with PGRs (particularly a combination of GA_3_ and cytokinins) followed by transfer to ammonium-containing medium with and then without PGRs [107], but this hypothesis requires further investigation.

In conclusion, some cryopreserved plant materials can recover on medium without PGRs, but for the majority, the presence of PGRs and their composition at the recovery stage is vitally important. The type and concentration of PGRs depend on material type rather than the cryopreservation method or medium formulation and may evolve with the protocol optimization. In a few papers where the effects of PGRs were compared, BA and GA_3_ alone or in combination were best suited for the recovery of the shoot tips and supported direct shoot development while minimizing callus formation. An additional supply of auxins at concentrations 2- to 10-times lower than GA_3_ or BA was beneficial for some species. Cell cultures required a higher level of auxins 2,4-D or NAA alone (undifferentiated cell cultures) or in balance with cytokinins (embryogenic cultures) for regrowth. Root culture requirements varied from no PGRs for hairy roots to auxins for adventitious roots and GA_3_, BA, or kinetin for shoot development from root sections. Notably, most cryopreservation protocols utilize relatively low levels of PGRs (up to 1.0 mg L^−1^ in most cases), perhaps as a way to reduce the chances for somaclonal and genetic variation in the recovering tissues.

## 7. Strategy 5. Light/Dark Conditions and Light Quality

Physical conditions in the room where the tissues are placed after rewarming should also be thoroughly considered. In particular, light quantity and quality, i.e., its intensity, photoperiod, and spectral composition, may dramatically affect both explant recovery and the morphogenic response [242]. It is generally accepted that, immediately after cryopreservation, the propagules require incubation in darkness to reduce the accumulation of potentially harmful photo-oxidative effects, particularly ROS [24,30]. The optimum period of “no light” varies depending on species and the type of propagules from 1 to 30 and even 60 days [24]; but 3 to 10 days are the most common [30]. For example, dark conditions were used for 7 days during the recovery of cryopreserved shoot tips of *Saccharum officinarum* [243] and *Ananas comosus* [244], *Citrus* somatic embryos [12], as well as orchid rhizomes and protocorms [245], while 4–5 days in the dark was enough for shoot tips of the American elm [129] and cassava [246], and root segments of *Hypericum perforatum* [212]. Darkness for only 3 days was sufficient for adventitious buds of lily hybrid [218].

After the period of darkness, the return to normal light conditions may be done step-wise by exposing the survived propagules to low-intensity dim light, usually varying from 5 to 16 μmol m^−2^s^−1^. For example, garlic shoot tips cryopreserved at the USDA-ARS National Plant Germplasm System were placed in the dark immediately after cryopreservation for 6 days, followed by dim light (16 h photoperiod, 6 µmol m^−2^s^−1^) for another 5 days and finally exposed to a growth chamber light (42 μmol m^−2^ s^−1^, 16 h photoperiods) [70]. Likewise, cryopreserved shoot tips of taro (*Colocasia esculenta* var. *esculenta*) were maintained in the dark for 5 days and then transferred to dim light (3.5 μmol m^−2^ s^−1^) for 10 days before exposure to normal light at 30 μmol m^−2^ s^−1^ [64].

However, the dark adaptation period is not always necessary. Dim light (5 μmol m^−2^ s^−1^) for the initial 5 or 7 days after rewarming was used for the successful recovery of, respectively, *Betula lenta* and *Holostemma annulare* shoot tips [104,183]. The large-scale cryopreservation program for *Allium* spp. at the National Agrobiodiversity Center (Republic of Korea) employed recovery under dim light (25 μmol m^−2^ s^−1^, 16 h photoperiod) as a part of a standard procedure [6]. Cryopreserved potato shoot tips could also recover under diffuse (6–15 μmol m^−2^ s^−1^) [241,247] and even intensive (104 μmol m^−2^ s^−1^, [248]) light with shoot regeneration reaching 30 to 100% depending on the protocol employed. However, later reports suggested that the cryopreserved potato shoot tips should be kept in the dark for the first 3–7 days for better recovery [57,249]. The current program for the large-scale potato cryopreservation at CIP employs recovery for 9 days in darkness followed by 4 days under diffuse light created by covering the top of the Petri dishes with a sheet of aluminum foil and then the normal light intensity of 80-100 μmol m^−2^s^−1^ [63]. Köpnick et al. [48] compared three protocols for cryopreservation of potato shoot tips: droplet freezing with dimethyl sulfoxide (DMSO) and PVS3-based droplet-vitrification procedure in two modifications. These methods also implied different conditions for recovery: 16 h low light (5 μmol m^−2^ s^−1^) for 7 days in the DMSO-based protocol and 7 days of darkness for two vitrification-based protocols. This is an example of how well-established protocols may also have different standard conditions for recovery.

When light and dark conditions for recovery were compared in the same study, the best recovery percentage seemed to be often achieved in the dark. For example, the survival of *Garcinia mangostana* shoot tips significantly increased to 50% when recovered for 7 days in the darkness before transferring to 16 h photoperiod compared to 7% of shoot tips recovered under normal light [250]. Cryopreserved shoot tips of *Saccharum officinarum* showed 100% regrowth when kept in the dark for 7 days compared to 56.7% regrowth of those maintained under light [243]. At the same time, Bespalova et al. [251] found no significant improvement in post-cryogenic regeneration in potato shoot tips recovered in darkness for 7 days compared to recovery under a 16 h photoperiod in three out of four tested genotypes. No correlation was found between explant type (shoot tips or axillary buds) and recovery conditions (dark/16 h photoperiod). Downey et al. [252] experimented with the duration of dark/gradual light/ambient light periods to recover cryopreserved cannabis (*Cannabis sativa*) shoot tips. They found that the variants “darkness (10 days)-gradual light (5 days)-ambient light (15 days)” and “darkness (14 days)-gradual light (7 days)-ambient light (9 days)” produced similarly high regrowth above 50%. Removing the gradual light step reduced the regrowth. The lowest regrowth percentage (ca. 27%) was observed when the initial dark step was expended to 20–21 days combined with a short normal light period (2–5 days). These findings confirm the earlier observations [247] that timely transfer of the recovering shoot tips to a normal light may be critical for normal plant regeneration in some species.

The modification of light spectra both before and after cryopreservation may also improve survival and recovery [30]. The advances in light-emitting diode (LED) technology are often used to control growth and morphogenesis during plant micropropagation [242], which can be helpful in both pre-LN and post-LN treatments. Edesi et al. [253] tested the effect of light quality, including fluorescent light, warm white light, blue LED, red LED, red with 10% blue, and red with 10% blue plus 20% of far-red LED, on the post-cryogenic recovery rate of potato shoot tips. They reported that red light with 10% of blue (LED) improved the regeneration percentage by 27% for one cultivar and by ca. 18% for the other four cultivars tested. For one of the cultivars, regrowth was improved from 0 to 17%, which meant a breakthrough for its potential cryopreservation upon further protocol optimization [253]. However, when different light spectra were applied before cryopreservation, the best recovery was observed following blue light treatment [175]. A similar study performed on cryopreserved root segments of *Hypericum perforatum* also demonstrated that red light or a combination of red and blue lights for the first 20 days of recovery improved production of shoots to 20–30%, which was significantly higher than constant darkness (5% regeneration) or fluorescent light (13% regeneration). Blue light (LED) alone could not support shoot regrowth [254].

The mechanism by which light exposure and particularly light spectra influence the recovery and subsequent plant regeneration of cryogenically exposed propagules is still obscure. In view of cryo-related injuries (discussed above) and generally low photosynthetic activity of in vitro cultured plants, one could hypothesize that photosynthetic reactions in green tissues should be strongly inhibited after cryopreservation. Bukhov et al. [255] demonstrated that the vitrification-based cryopreservation of *Bratonia* orchid protocorms caused disorders in linear electron transport between photosystems II and I (PSII and PSI), most likely due to the disruption of the functional connection of electron carriers between the plastoquinone pool and the PSI reaction center. However, immediately after rewarming, some photosynthetic activity in protocorms was still registered, and alternative electron transport pathways related to photosystem I were functioning [255]. The authors noted that mobilization of PSI-driven alternative electron transport pathways might be essential to cope with the deficiency of energy equivalents in phototropic cells subjected to stress factors. The ability for P700 photooxidation was lost after only 24 h of regrowth, possibly due to the progressive development of the injuries in thylakoid membranes and other cell structures in the non-optimized cryopreservation protocol, eventually leading to protocorm death within several days. It is, therefore, possible that photosynthetic reactions may have at least some impact on energy provision for regrowth.

Some answers can be drawn from publications not related to cryopreservation. For example, the combination of red and blue lights that showed promising results in improving post-cryopreservation regrowth was also reported to improve organogenesis, promote differentiation, proliferation and growth [256], induce the synthesis of new protein bands [242] and secondary xylem formation [257], reduce hyperhydricity [258], increase the content of soluble carbohydrates, and free amino acids in shoots [242]. Red LED light can also lower the concentration of endogenous phenolic compounds in plantlets [259]. All of these actions potentially benefit plant tissues after cryopreservation as there is a need to repair and retain their morphogenic potential. Working with leaf explants of *Curculigo orchioides* in vitro, Gupta and Sahoo [260] found that red and blue LED lights and their combination induced changes in the intracellular level of ROS and demonstrated the complex interplay between the activities of antioxidant machinery enzymes (CAT, APX, SOD, peroxidase, glutathione reductase) and light quality [260]. These studies also suggest that the mechanism of light quality effect on the recovery may also be related to signaling pathways.

Thus, the role of light/dark conditions after cryopreservation is not straightforward and requires further investigation. While most propagules benefit from the recovery taking place in darkness, light seems critically essential for the morphogenesis, regeneration, and growth of healthy shoots, and these processes are strongly affected by light quality. Based on our analysis of the literature, it is also possible that plant materials with higher sensitivity to osmotic and dehydration stress provoked by cryopreservation require a more extended period of darkness recovery compared to less sensitive explants.

## 8. Conclusions and Prospects

Though cryopreservation is a unique option for the long-term conservation of vegetatively propagated crops, ornamental and threatened species with non-orthodox seeds, it is limited by a complex methodology that requires time-consuming and laborious optimization and the low-to-moderate regrowth percentage of plant material that is sensitive to cryopreservation stress. Even when cryopreserved through optimized protocols, plant organs and cells experience extensive damages, both reversible and irreversible, and require an exceptional nourishing environment to support recovery and regrowth.

**Figure 2:**
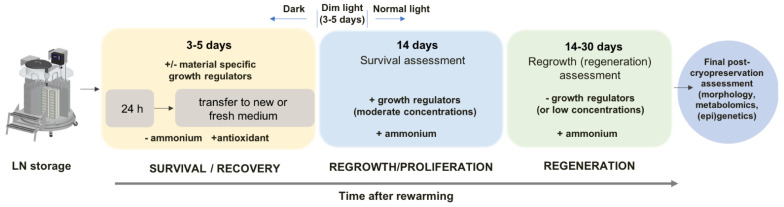
Potential combinations of various strategies at the post-LN steps for plant materials sensitive to cryopreservation-induced stresses. LN—liquid nitrogen.

Different strategies can be combined at the post-LN stage to support the rapid recovery of plant material (Figure 2). The post-LN recovery of cryopreserved materials consists of two basic steps: recovery *per se,* i.e., the first several days after cryopreservation when the cells struggle for survival in response to the extensive damage, and the regrowth step, i.e., when the surviving cell groups start to proliferate intensively and give rise to cell mass or new morphological structures (new roots, shoots or embryos depending on the type of the cryopreserved tissues). These two steps require the use of different strategies.

The step-wise increase of medium osmotic potential and the use of a solid medium may help to cope with the rapid change in the osmotic environment. Exogenous additives with ROS-scavenging activities mitigate the oxidation stress, while others absorb toxic chemicals released from the damaged tissues. Iron-free and/or ammonium-free medium may be necessary for sensitive materials during the initial days after rewarming. Still, these ions are essential for the following steps to complete the regrowth process. Plant growth regulators play a significant role in the fast and normal development of the recovered tissues. For shoot tips and other propagules containing shoot meristems, BA and GA_3_ alone or in combination with low concentrations of auxins provide better responses. Undifferentiated cell cultures typically require moderate concentrations of 2,4-D or NAA in recovery medium, while somatic embryos, orchid protocorms, and hairy root tips can recover on medium without PGRs. Adventitious buds, rhizome buds, and embryogenic cultures show better responses on medium with a balanced combination of auxins and cytokinins. Although darkness is generally recommended during the initial recovery, the duration of dark culture is species-dependent, and a timely transfer to normal light is critically important for normal shoot development from the survived meristems. Moreover, a combination of red and blue light seems beneficial during recovery, although further tests are required.

In stress-sensitive genotypes, step-wise recovery after cryopreservation seems to be particularly important. The first step may combine dark recovery on solid, ammonium-free medium, or full-strength medium with the addition of antioxidant compounds for 5–7 days, followed by full-strength medium with growth regulators selected depending on material type. Dim light can be provided after days 5–7 for several days, followed by regular light. One more transfer to medium without growth regulators may be necessary for plant development after 2–3 weeks. These considerations are general, as the recovery process still needs to be tailored to the requirements of the specific material. At the same time, we believe that a better understanding of the regrowth patterns discussed above will help researchers choose the most effective procedures for new materials that need to be cryobanked.

## Figures and Tables

**Table 1:** Examples of cryopreservation studies using ammonium-free regrowth medium.

Species (Material)	Method *	Pre-LN Conditions **	Regrowth Conditions	Regrowth (R), Regeneration (RG) (Change from Ammonium-Containing Medium) ***	References
**Comparison of ammonium-containing and ammonium-free medium**					
*Oryza sativa * (cells)	SF	S-2% (24 h) → S-10% (24 h); D-glucose 20% + DMSO 10% (added gradually during 30 min + 30 min for equilibration, ice); 1°C/min to −30°C	+NH_4_NO_3_, 10^−5^M 2,4-D, 7 d	TTC: 6.5%	[92]
			−NH_4_NO_3_, 10^−5^M 2,4-D, 7 d	TTC: 27.5% (+21%)	
*Lavandula vera* (cells)	SF	D-glucose 20% + DMSO 10% (added gradually during 30 min + 30 min for equilibration, ice); 1°C/min to −40°C	+NH_4_NO_3_, 10^−5^ M 2,4-D, 7 d	TTC: 0.1 (absorbance)	[101]
			−NH_4_NO_3_, 10^−5^ M 2,4-D, 7 d	TTC: 0.38 (+3.8x) (absorbance)	
*Oryza sativa * (cells)	SF	S-13.4%; glycerol 10% + DMSO 10%; 1°C/min to −40°C	+NH_4_NO_3_, 3d	TTC reduction: 0.8 (absorbance)	[102]
			−NH_4_NO_3_, 8 h → +NH_4_NO_3_, 3 d	TTC reduction: 2.4 (+3x) (absorbance)	
*Betula pendula* (shoot tips)	SF	ABA 100μM + DMSO 0.5% (3 d, 5°C); D-glucose 20% + DMSO 10% (added gradually during 30 min + 30 min for equilibration, ice); 10°C/h to −38°C	+NH_4_NO_3_, + Ca(NO_3_)_2_, 3 d	R: 0%	[103]
			−NH_4_NO_3_/ +KNO_3_ + Ca(NO_3_)_2_ at cold hardening, cryoprotection, unloading, recovery, 3 d	R: 53.0% (+53.0%)	
			−NH_4_NO_3,_ −Ca(NO_3_)_2_ /+KNO_3_ at cold hardening, cryoprotection, unloading and recovery, 3 d	R: 58.3% (+58.3%)	
*Betula pendula* (shoot tips from young 20-month cultures)	SF	ABA 100μM + DMSO 5% (3 d, 5°C); polyethylene glycol 10% + D-glucose 10% + DMSO 10% (30 min, 0°C); 0.17°C/min to −38°C	+NH_4_NO_3_ 5 mM, +Ca(NO_3_)_2_ 2mM, 3 d	R: 37.5%	[91]
			−NH_4_NO_3_, −Ca(NO_3_)_2_/+KNO_3_ 10mM at cold hardening, cryoprotection, unloading, recovery, 3 d	R: 53.7% (+16.2%)	
*Ipomoea batatas* (shoot tips)	VT	S-2% (24 h) → S-10% (24 h); glycerol 18.4% + S-20.5% (60 min); PVS2 (16 min, RT)	+NH_4_NO_3_, 2.2 μM BA + 0.49 μM IBA, 5d → MS, ~8 w	RG: 32%	[96]
			−NH_4_NO_3_, 2.2 μM BA + 0.49 μM IBA, 5d → MS, ~8 w	RG: 93% (+61%)	
*Holostemma annulare* (shoot tips)	ED	S-17.1% (24 h) → S-25.6% (24 h); S-25.6% + DMSO 3% (3 d, 4°C); Air drying to 0.17–0.2 g water/g DW	+NH_4_NO_3_, 45 d	R: 7.7~22.7%	[93]
			−NH_4_NO_3_, 45 d	R: 34.2~58.6% (+26.5~35.9%)	
*Holostemma annulare* (shoot tips)	ED	S-17.1% (24 h) → S-25.6% (24 h); S-25.6% + DMSO 3% (3 d, 4°C); Air drying to 0.17–0.2 g water/g DW	+NH_4_NO_3_ 20.6 mM all through the process including recovery (45 d)	R: 12%	[104]
			Reduced NH_4_NO_3_ (2.6mM) during preparative culture and preconditioning; −NH_4_NO_3_ at preculture and recovery (45 d)	R: 55% (+43%)	
*Bletilla striata* (protocorms)	DV	S-17.5% (3 h); glycerol 18.2% + S-13.4% (15 min); PVS2 (40 min, 25°C)	+NH_4_NO_3_ 480 mg L^−1^, 2 months	R: 44~66%	[105]
			−NH_4_NO_3_, 2 months Too long on medium without ammonium?	R: 11~32% (−33–34%)	
*Dioscorea alata * (shoot tips)	VT	S-17.5% (3 h); glycerol 18.2% + S-13.4% (15 min); PVS2 (40 min, 25°C)	+NH_4_NO_3_ 1650 mg L^−1^, 40 d	RG: 32.2%	[100]
			+NH_4_NO_3_ 330 mg L^−1^, 40 d	RG: 35.6~38.9% (+3.4~6.7%)	
*Ipomoea batatas* (shoot tips)	DV	S-10% (31 h) → S-17.5% (17 h); C4-35% (50 min); PVS3 (60 min, RT)	−NH_4_NO_3_, 1 mg L^−1^ GA_3_ + 0.5 mg L^−1^ BA, 1w → +NH_4_NO_3_, 0.5 mg L^−1^ BA, 4w (total 5 w)	RG: 21.0%	[97]
			−NH_4_NO_3_, 1 mg L^−1^ GA_3_ + 0.5 mg L^−1^, 1w → +NH_4_NO_3_, 0.5 mg L^−1^ GA_3_, 4w →+NH_4_NO_3_, 0.5 mg L^−1^ GA_3_, 3w (total 2 months)	RG: 56.0% (+35.0%)	
			−NH_4_NO_3_, 1 mg L^−1^ GA_3_ + 0.5 mg L^−1^, 1w → +NH_4_NO_3_, 0.5 mg L^−1^ GA, 3w → MS, 4 w) (total 3 months)	RG: 67.5% (+46.5%)	
*Osmunda regalis,* 2 varieties (gametophyte)	EV	S-8.6% (2 weeks); glycerol 18.2% + S-13.4% (20 min); PVS3 (3 h, RT)	+NH_4_NO_3_ in 1/2, 1/4 or 1/8 MS medium, 6w (comparison in fresh control only)	Proliferation of fresh control: 71~78%	[106]
			−NH_4_NO_3_ and vitamin-free in MS medium, 6w (comparison in fresh control only)	Proliferation of fresh control: 83~89% (+11~12%)	
*Chrysanthemum morifolium*, 2 varieties (shoot tips)	DV	S-10% (30 h) → S-17.5% (16 h); C6-40% (30 min); B5-80% (60 min, RT)	+NH_4_NO_3_, 2w→ MS, 2w	RG: 38.8~42.1%	[95]
			−NH_4_NO_3_, 2w→MS, 2w	RG: 75.5~80.7% (+36.7~38.6%)	
*Citrus limon*, 2 varieties (shoot tips)	DV	S-10% (48 h) → S-17.5% (16 h); C4-35% (40 min); PVS2 (60 min, 0°C)	+NH_4_NO_3_, 1d → MS, 1w → grafting	RG: 50.3~53.5%	[94]
			−NH_4_NO_3_, 1d or 1/4 NH_4_NO_3_, 1d →MS, 1w → grafting	RG: 67.3~70.3% (+16.8~17.0%)	
*Aster altaicus* var. *uchiyamae* (shoot tips)	DV	S-10% (55 h) → S-17.5% (17 h); C4-35% (60 min, ice); A3-80% (60 min, ice)	+NH_4_NO_3_, hormone-free, 4w → MS	RG: 31.0%	[98]
			−NH_4_NO_3_, 1 mg L^−1^ GA_3_ + 0.5 mg L^−1^ BA, 5d→ +NH_4_NO_3_, 0.5 mg L^−1^ GA_3_, 3w2d → MS	RG: 64.0% (+33.0%)	
*Pogostemon yatabeanus* (shoot tips)	DV	S-10% (31 h); C4-35% (40 min); A3-80% (60 min, ice)	+NH_4_NO_3_, 1 mg L^−1^ GA_3_ + 0.5 mg L^−1^ BA, 5d → +NH_4_NO_3_, 1 mg L^−1^ GA_3_ + 0.5 mg L^−1^ BA, 3w2d→ MS	RG: 63.3%	[107]
			−NH_4_NO_3_, 1 mg L^−1^ GA_3_ + 0.5 mg L^−1^ BA, 5d → +NH_4_NO_3_, 1 mg L^−1^ GA_3_ + 0.5 mg L^−1^, 3w2d → MS	RG: 95.7% (+ 32.4%)	
**Use of ammonium-free medium without comparison**					
*Fragaria x ananassa* var. *“Massey”*, “MDUS3816” (shoot tips)	DV	S-10% (30 h) → S-17.5% (16h); C4-35% (40 min); PVS3 (60 min, RT)	−NH_4_NO_3_, 1 mg L^−1^ GA_3_ + 0.5 mg L^−1^ BA, 5w → +NH_4_NO_3_, 0.5 mg L^−1^ GA_3_, 9w	R: 65.5% (var. “Massey”) R: 50.0% (var. “MDUS3816”)	[108]
*Fragaria x ananassa* var. “Wonkyo3114”, “Gurumi40”	DV	S-10% (40 h); C4-35% (40 min); B5-80% (40 min, RT)	−NH_4_NO_3_, 1 mg L^−1^ GA_3_ + 0.5 mg L^−1^ BA, 5w → +NH_4_NO_3_, 0.5 mg L^−1^ GA_3_, 9w	R: 55.6% (var. “Wonkyo3114”) R: 50.5% (var. “Gurumi40”)	
*Malus domestica* 5 varieties (shoot tips)	VT	S-25% 5°C, 1 d; PVS2 (80 min, RT)	−NH_4_NO_3_	R: 45.0~77.5%	[109]
*Pyrus* spp., 8 varieties (shoot tips)	VT	S-25% 5°C, 1 d; PVS2 (80 min, RT)	−NH_4_NO_3_	R: 40.0~70.0%	[109]
12 *Vitis* species	DV	S-10% + 0.1 mM salicylic acid + 1 mM ascorbic acid +1 mM glutathione (reduced form) (3 d); glycerol 18.2% + S-13.4% (20 min); ½ PVS2 (20 min) → PVS2 (60–105 min, 0°C)	−NH_4_NO_3_, S-20.5%, overnight → −NH_4_NO_3_, 0.2 mg L^−1^ BA, 2 w → +NH_4_NO_3_, 0.2 mg L^−1^ BA	R: 37–53%	[49]

* SF, slow freezing; VT, vitrification; ED, encapsulation-dehydration; EV, encapsulation-vitrification; DV, droplet-vitrification; RT, room temperature; DW, dry weight; d, days. ** DMSO, dimethylsulfoxide; S, sucrose; C4-35%, 17.5% glycerol + 17.5% sucrose; C6-40%, 20% glycerol + 20% sucrose; PVS2: 30 % (*w*/*v*) glycerol + 15 % (*w*/*v*) DMSO + 15 % (*w*/*v*) ethylene glycol + 0.4 M sucrose; A3-90%: 37.5 % glycerol + 15 % DMSO + 15 % ethylene glycol + 22.5 % sucrose, *w*/*v*; A3-80%: 37.5 % glycerol + 15 % DMSO + 15 % ethylene glycol + 22.5 % sucrose, *w*/*v*; PVS3: 50% (*w*/*v*) glycerol + 50% (*w*/*v*) sucrose; B5-80%: 40% (*w*/*v*) glycerol + 40% (*w*/*v*) sucrose. *** Mean regrowth (R) and regeneration (RG) after cryopreservation using the best protocol reported. Definition of “Regrowth” was variable in the literature cited. It indicated size increase in cryopreserved explants and/or the formation of new roots and/or shoots. “Regeneration” indicated development of normal plantlets with elongated stems, leaves and roots. TTC, triphenyl tetrazolium chloride; w, weeks.

**Table 2:** The use of the exogenous compounds with antioxidant and detoxifying potential at the recovery step after cryopreservation of different plant species and material types.

Exogenous Compound	Proposed Action	Effective Concentration	Species Tested	Material Cryopreserved	Effectiveness	Reference
Ascorbic acid	Antioxidant	0.14–0.58 mM	*Rubus,* 2 varieties	Shoot tips	Up to 35% improvement	[85]
		10–50 µM	*Paphiopedilum insigne*	Protocorms	Little or no improvement *	[76]
		0.6 mM	*Dendrobium* so nia-28	Protocorm-like bodies	∼8–11% improvement after 6 weeks; stable improvement by ∼15% when combined with 2 g L^−1^ activated charcoal	[156]
Vitamin E **	Antioxidant	11 and 15 mM	*Rubus,* 2 varieties	Shoot tips	Up to 25% improvement	[85]
Tocopherol	Antioxidant	10–50 µM	*Paphiopedilum insigne*	Protocorms	Little or no improvement *	[76]
Lipoic acid	Antioxidant	2–10 mM (optimum 2–6 mM)	*Rubus* spp.	Shoot tips	∼10% improvement	[157]
Glutathione, reduced form (GSH)	Antioxidant	0.08–0.33 mM	*Rubus* spp.	Shoot tips	10–25% improvement	[157]
		10–50 µM (optimum 20–30 μM)	*Paphiopedilum insigne*	Protocorms	Regrowth improved by ∼10–25% *	[76]
		32.5 μM	*Citrus* spp.	Shoot tips	~7% Survival improvement	[158]
Glycine betaine	Antioxidant	5–20 mM (optimum 10 mM)	*Rubus* spp.	Shoot tips	∼26% improvement	[157]
Melatonin	Antioxidant, signaling, regulation of metabolic pathways	0.1 or 0.5 µM	*Ulmus americ* *ana*	Shoot tips	∼25% improvement	[129]
		0.1 µM	*Dioscorea alata* and *D. cayenensis*	Shoot tips	Up to 20% regeneration improvement	[159]
		0.1–0.5 µM	*Nicotiana tabacum* *Hypericum perforatum*	Shoot tips	30–40% regrowth improvement	[160]
Desferrioxamine	Iron sequestration, prevention of harmful Fenton and free radical cascade reactions	0.5 and 10 mg L^−1^	*Oryza sativa* cv. Taipei 309	Embryogenic cell culture	Improved cell viability measured by TTC. Higher weight gain (by 20–30%)	[84]
Phloroglucinol	Antioxidant	10–50 µM (optimum 30 μM)	*Paphiopedilum insigne*	Protocorms	∼23% regrowth improvement *	[76]
Polyvinylpyrrolidon	Antioxidant	1–10 mM	*Rubus* spp.	Shoot tips	Negative effect	[157]
Pluronic F-68	Nonionic surfactant, protection against fluid-mechanical damage, promote cell division	0.005%	*Solanum tuberosum*	Shoot tips	16–33% plant regeneration improvement	[161]
		0.01%	*Oryza sativa* cv. Taipei 309	Embryogenic cell culture	36% cell viability improvement	[162]
Pluronic F-68 + oxygenated perfluorocarbon (OP)	Improve oxygen transfer, promote cell division	0.01%	*Oryza sativa* cv. Taipei 309	Embryogenic cell culture	20–24% cell viability improvement	[162]
Oxygenated perfluorocarbon (OP)	Improve cell proliferation, increase oxygen transfer rates	Semi-solid medium overlaying 20.0-mL aliquots of OP	*Oryza sativa* cv. Taipei 309	Embryogenic cell culture	57% cell viability improvement	[162]
Gold nanoparticles (AuNPs)	Shift in antioxidant enzyme activities observed	10–30 ppm	*Lamprocapnos spectabilis*	Shoot tips	Reduced survival (but improved when applied at pre-LN step)	[163]

* The exogenous substance was applied at both the pre-LN and post-LN step in the same protocol. ** Vitamin E was a mixture of tocopherols, tocotrienols and α-tocopheryl polyethylene glycol 1000 succinate. TTC—Triphenyl tetrazolium chloride

## Data Availability

All data have been collected from scientific publications available through open access or institutional subscriptions.
